# Multifunctional Small Molecules as Potential Anti-Alzheimer’s Disease Agents

**DOI:** 10.3390/molecules26196015

**Published:** 2021-10-03

**Authors:** Beatrice Bargagna, Lidia Ciccone, Susanna Nencetti, M. Amélia Santos, Sílvia Chaves, Caterina Camodeca, Elisabetta Orlandini

**Affiliations:** 1 Department of Earth Sciences, University of Pisa, Via Santa Maria 53-55, 56100 Pisa, Italy; beatrice.bargagna@dst.unipi.it; 2 Centro de Química Estrutural and Departamento de Engenharia Química, Instituto Superior Técnico, Universidade de Lisboa, Av. Rovisco Pais, 1049-001 Lisboa, Portugal; silvia.chaves@tecnico.ulisboa.pt; 3 Department of Pharmacy, University of Pisa, Via Bonanno 6, 56126 Pisa, Italy; lidia.ciccone@unipi.it (L.C.); susanna.nencetti@unipi.it (S.N.); caterina.camodeca@unipi.it (C.C.); 4 Research Center “E. Piaggio”, University of Pisa, 56122 Pisa, Italy

**Keywords:** Alzheimer’s disease (AD), multifunctional drugs, metal chelation, antioxidant activity, Aβ stabilizers, neurodegeneration, ferulic acid, multitarget drugs

## Abstract

Alzheimer’s disease (AD) is a severe multifactorial neurodegenerative disorder characterized by a progressive loss of neurons in the brain. Despite research efforts, the pathogenesis and mechanism of AD progression are not yet completely understood. There are only a few symptomatic drugs approved for the treatment of AD. The multifactorial character of AD suggests that it is important to develop molecules able to target the numerous pathological mechanisms associated with the disease. Thus, in the context of the worldwide recognized interest of multifunctional ligand therapy, we report herein the synthesis, characterization, physicochemical and biological evaluation of a set of five (**1a**–**e**) new ferulic acid-based hybrid compounds, namely feroyl-benzyloxyamidic derivatives enclosing different substituent groups, as potential anti-Alzheimer’s disease agents. These hybrids can keep both the radical scavenging activity and metal chelation capacity of the naturally occurring ferulic acid scaffold, presenting also good/mild capacity for inhibition of self-Aβ aggregation and fairly good inhibition of Cu-induced Aβ aggregation. The predicted pharmacokinetic properties point towards good absorption, comparable to known oral drugs.

## 1. Introduction

Alzheimer’s disease (AD) is a multifactorial neurodegenerative disorder characterized by a progressive loss of neurons in the brain. Early symptoms are memory decline and language problems, followed by other cognitive serious dysfunctions related to brain atrophy [1]. AD is the most common cause of dementia and, in 2019, it was estimated that 50 million individuals were affected by dementia worldwide. This number is projected to reach 152 million cases by 2050 [2]. Despite research efforts, the pathogenesis and mechanism of AD progression are not yet completely understood. However, it is well-known that a common feature in AD patients is the presence of extracellular amyloid-β (Aβ) plaques and intracellular neurofibrillary tangles (NFT) of hyperphosphorylated tau protein, the two major hallmarks in AD [3]. 

There are only a few symptomatic drugs approved for the treatment of AD. Four of them hamper the pathway that downregulates the neurotransmitter acetylcholine (ACh) acting as acetylcholinesterase inhibitors (AChE)—tacrine, donepezil, rivastigmine and galantamine—and the fifth is a *N*-methyl-d-aspartate (NMDA) receptor antagonist (memantine) [4]. Moreover, on 7 June 2021, the Food and Drug Administration (FDA) approved Aduhelm (aducanumab) for the treatment of AD. Aducanumab is a human IgG1 anti-Aβ monoclonal antibody specific for β-amyloid oligomers and fibrils [5].

The multifactorial character of AD suggests that it is important to develop molecules able to target the numerous pathological mechanisms associated with the disease. Studies revealed that Aβ is the most abundant peptide found among the numerous proteins present in AD amyloid plaques [6]. It has been demonstrated that these proteins can interact with Aβ peptides favoring or contrasting AD progression—negative and positive cross-interaction, respectively. Recently, the positive amyloid cross-interaction has been reported as a potential emerging multi-target strategy against AD [7,8] and several proteolysis targeting chimera (PROTAC) constructions have been proposed [9,10,11].

Dyshomeostasis of physiological metal ions is a common feature of neurological disorders such as AD [12,13,14,15,16,17,18,19]. Studies report that higher levels of metal ions, such as Cu^2+^, Zn^2+^ and Fe^3+^, are found in cerebral amyloid plaques of AD patients compared to the concentrations detected in the brains of non-AD patients [13,20]. Moreover, it has been reported that redox active metal ions, as Cu^2+^ and Fe^2+^, interact with Aβ producing reactive oxygen species (ROS) and, finally, inducing neuronal death [21]. Therefore, most chelators included in anti-AD drugs are based on heterocyclic structures of hard or hard-soft ligands, namely containing endocyclic or hexocyclic pairs of electron donor atoms such as (*O*-*O*), (*O*-*N*), and (*N*-*N*). According to the multifactorial nature of AD, the most recent studies aim to develop molecules that can act simultaneously against different pathological features, at the same time being Aβ stabilizers, antioxidants and metal chelators [22,23]. 

In the context of multifunctional ligand therapy, marine and terrestrial organisms are a fundamental source for the discovery of new bioactive agents [24,25,26,27,28]. In the last few years, several different compounds isolated from plants and microorganisms have shown good effects for the treatment of AD and several drug candidates are in clinical trials, confirming that the use of natural compounds against AD is an active and interesting area of research [23,29,30,31].

Ferulic acid (FA), as other hydroxycinnamic derivatives, is a phenolic compound largely present in the human diet. FA has been considered as a multifunctional antioxidant because, besides the more typical radical scavenging role by electron or hydrogen donation to existing radicals, it can also chelate redox-active metal ions, thus disabling their participation in the Fenton reaction. FA is also well known for its anti-inflammatory properties and recent studies have demonstrated its potential role in the treatment of AD, particularly due to its capacity to inhibit Aβ aggregation in vitro and in in vivo AD mouse models protecting the brain from Aβ neurotoxicity [32]. In fact, in the last few years, FA has been largely used as a scaffold to design new multifunctional ligands against AD progression [33].

**Figure 1:**
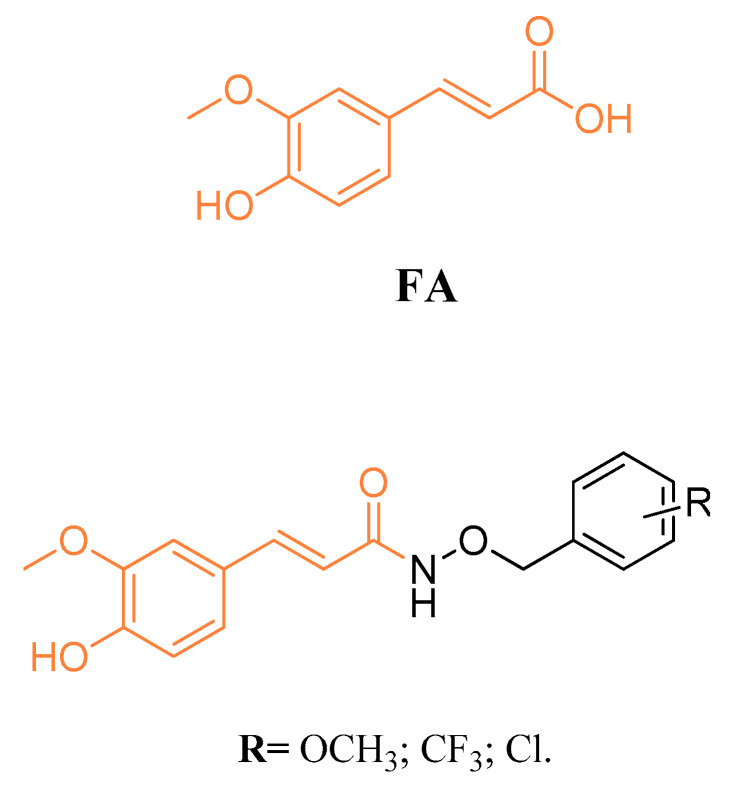
General structures of ferulic acid (FA) and its benzyloxyamidic derivatives.

Given the considerations above, in this study, a new set of FA hybrid derivatives, **1a–e**, is presented in which the FA moiety is coupled with benzyloxyamines substituted with methoxyl and trifluoromethyl groups or also chlorine atoms (Figure 1). In particular, the methoxyl and trifluoromethyl groups were chosen for their ability to establish H-bond interactions as acceptors or donors, or only as acceptors, respectively. Concerning the chlorine atom, it increases the lipophilic character of the molecule, thus favoring hydrophobic interactions with Aβ peptides. 

Herein, we report the synthesis and characterization of a new set of hybrid compounds enclosing the ferulic acid scaffold, followed by the evaluation of their physicochemical and biological properties, envisaging their potential role as anti-AD agents.

## 2. Results and Discussion

### 2.1. Chemistry

**Scheme 1:**
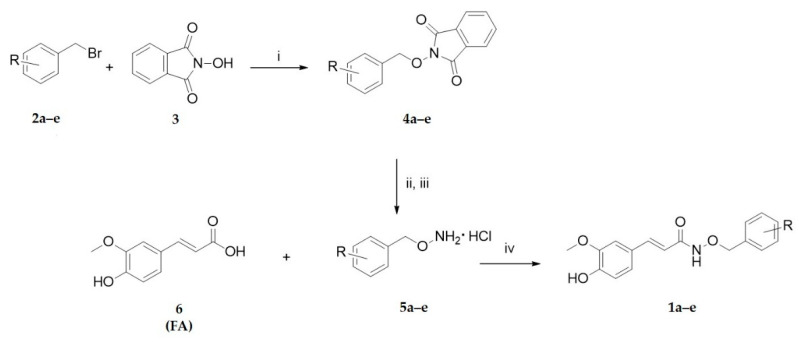
(i) Et_3_N, anhydrous DMF, r.t., 4 h; (ii) NH_3_ 7M in MeOH, r.t., 2 h; (iii) Et_2_O·HCl, Et_2_O, *T* = 0 °C; (iv) EDCI, HOBt, *N*-methylmorpholine, anhydrous DMF, r.t., 24 h.

The (*E)*-*N-*(benzyloxy)-3-(4-hydroxy-3-methoxyphenyl)acrylamide compounds, **1a**–**e**, were obtained following the synthetic procedure reported in Scheme 1.

The FA derivatives **1a**–**e** were synthesized coupling the commercially available ferulic acid (**6**) and hydrochloride benzylhydroxylamines **5a**–**e** variously substituted. The *O*-arylmethylhydroxylamine hydrochloride **5a**–**e** were synthetized according to the procedure previously described [34,35,36]. Briefly, the *O*-arylmethylhydroxylamine hydrochloride **5a**–**e** were obtained by reaction between the suitably substituted benzyl bromide **2a**–**e** and the *N*-hydroxyphthalimide (**3**) by Mitsunobu reaction, and successive deprotection of the phthalimido group with ammonia solution 7N in MeOH. Compounds **5a**–**e** were purified by crystallization and isolated as their hydrochloride salts.

Finally, the coupling reaction of the free amino group of these benzylhydroxylamines with the carboxylic group of FA was carried out in anhydrous DMF and inert argon atmosphere, in the presence of the carboxyl activating agent *N*-(3-dimethylaminopropyl)-*N*′-ethylcarbodiimide hydrochloride (EDCI), hydroxybenzotriazole (HOBt) and *N*-methylmorfoline. The reaction mixture was stirred at r.t. for 24 h, followed by the corresponding workup and purification to afford the final compounds **1a–e** as pure solids under good yields (57–69%).

### 2.2. Physicochemical Studies

#### 2.2.1. Antioxidant Activity

Cinnamic acid derivatives, such as FA, have been shown to avoid chain-breaking in the oxidation of low density lipoproteins, related with their hydrogen or electron-donating capacity and to the stability of the formed phenoxyl radicals [37].

Commercial ferulic acid (FA) and all the newly synthesized compounds **1a**–**e** were studied for their radical scavenging activity, following the protocol previously reported [38,39]. The activity of each compound is expressed as EC_50_ and is related to its interaction with the free stable radical 2,2-diphenyl-1-picrylhydrazyl (DPPH•). Analysis of the results contained in Table 1 shows that benzyloxyamidic derivatives **1a**–**e** have good antioxidant activity, similar to the one of ferulic acid and slightly lower than the one of ascorbic acid [40], with an EC_50_ value in the order of low µM. The transformation of the carboxylic portion of ferulic acid and the increasing size of the molecule do not affect their radical scavenging capacity, which must be related to the proton donor phenolic group, both in the precursor and in the hybrids.

#### 2.2.2. Metal Chelation Studies

Besides antioxidant capacity, FA can also play other roles such as chelation of transition metal ions (e.g., copper, iron), which are catalysts of oxidative stress, and interference in metal-induced Aβ aggregation. 

In order to evaluate the chelation capacity of the herein developed FA derivatives, compounds **1a** and **1d** were selected to be investigated on their acid-base behavior and metal chelating ability towards Cu^2+^ and Fe^3+^ ions, by using UV–Vis spectrophotometric titrations. These results were further compared to those of **FA**, studied by pH-potentiometric titrations. 

##### Acid-Base Properties 

To evaluate the metal complexation capacity of the selected compounds, their protonation constants were determined. 

Compounds were obtained in their neutral form, H_2_L (**FA**) and HL (**1a**, **1d**), respectively. Due to some water-solubility limitations, especially for the benzyloxyamidic derivatives **1a** and **d**, a mixed (20%, *w*/*w*) DMSO/water medium was chosen. The values obtained for the protonation constants are reported in Table 2. 

**Figure 2:**
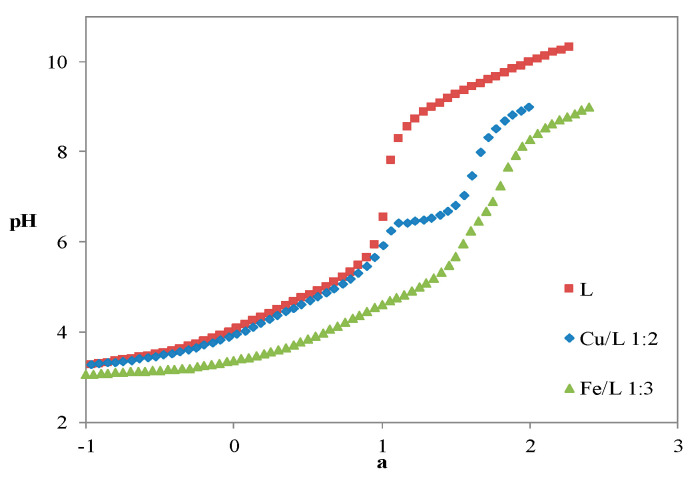
Potentiometric titration curves of **FA** in 20% DMSO/water medium in the absence or in the presence of Cu^2+^ and Fe^3+^ (*C*_L_ = 5-6.7 × 10^−4^ M, **a** represents moles of added base per mole of ligand).

The values were obtained by fitting analysis of the experimental pH-potentiometric (**FA**) and spectrophotometric data (**1a**, **1d**) with an equilibrium model using Hyperquad 2008 [41] and Psequad [42] programs, respectively. Figure 2 includes the potentiometric titration curves obtained for **FA**, as an example. 

The protonation constants calculated for ferulic acid (**FA**) and depicted in Table 2 are in accordance with values previously reported (log *K*_1_ = 8.77–8.94, log *K*_2_ = 4.46–4.56) [43,44,45], taking into consideration that the literature values were determined in aqueous medium and/or different ionic strength conditions. These constants correspond to the protonation of the phenolic and carboxylic groups of **FA**, respectively. Table 2 also encloses the protonation constants corresponding to the phenolic oxygen atom of **1a** (8.75) and **1d** (8.93), determined by spectrophotometric titration, which agree with log *K*_1_ of **FA**, as expected. The protonation constants corresponding to the NH amidic group of compounds **1a** and **1d** could not be determined because the NH_2_^+^ group is extremely acidic (typically p*K*_a_ < 0). In fact, due to the presence of the neighbor carbonyl group, the lone pair of electrons is no longer localized on the nitrogen atom and so the basicity of this center is quite reduced.

**Figure 3:**
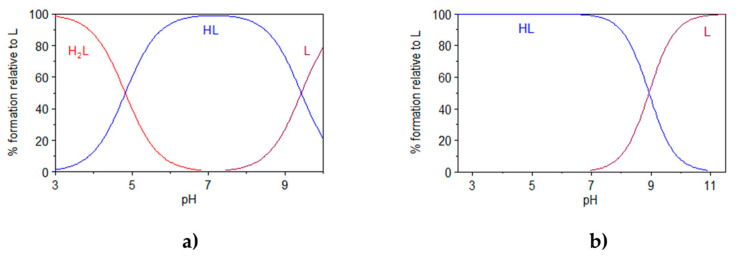
Species distribution curves for compounds (**a**) **FA** (*C*_L_ = 5 × 10^−4^ M) and (**b**) **1d** (*C*_L_ = 4.0 × 10^−5^ M).

Examples of species distribution curves, determined at the experimental conditions used in the pH-potentiometric and spectrophotometric titrations, are shown in Figure 3. The predominant species at assumed physiological conditions, pH 7.4 and concentration *C*_L_ = 10^−5^ M, are HL^−^ for **FA** (ca. 99%) and the neutral HL for **1a** (96%) and **1d** (97%). The existence of the neutral HL species in extremely high concentrations (96–97%) explains the need to use a mixed 20% *w/w* DMSO/water medium in the solution studies, as the lipo-hydrophilic character is not only determined by the molecular charge but also by solute–solvent interactions.

##### Metal Complexation 

The chelating ability of **FA** and of selected compounds **1a** and **1d** was studied by using the same experimental techniques (potentiometry, UV–Vis spectrophotometry) and experimental medium used for the titration of the ligand alone (20% *w/w* DMSO/water). In the following calculations for the 1:1, 1:2 and 1:3 metal/ligand (M/L) systems (M = Fe, Cu), the log *K*_i_ values previously obtained by each experimental method were used in the equilibrium model of the complexation studies performed by the same methodology.

Alterations in the deprotonation profiles of the ligand titration curves due to the presence of the metal ions are evident in Figure 2, since the curves for the M/L systems appear well below that of **FA** for **a** > −1 (M = Fe) or **a** > 0 (M = Cu). This evidence supports the formation of metal complexes with the deprotonated (L^−^) and mono-protonated (HL) forms of the ligand, whose stability order (Fe > Cu) follows the expected trend. Equilibrium models obtained from the fitting analysis of the potentiometric curves (M = Fe, Cu) and the UV–Vis spectral data (M = Fe, Cu) are reported in Table 2.

**Figure 4:**
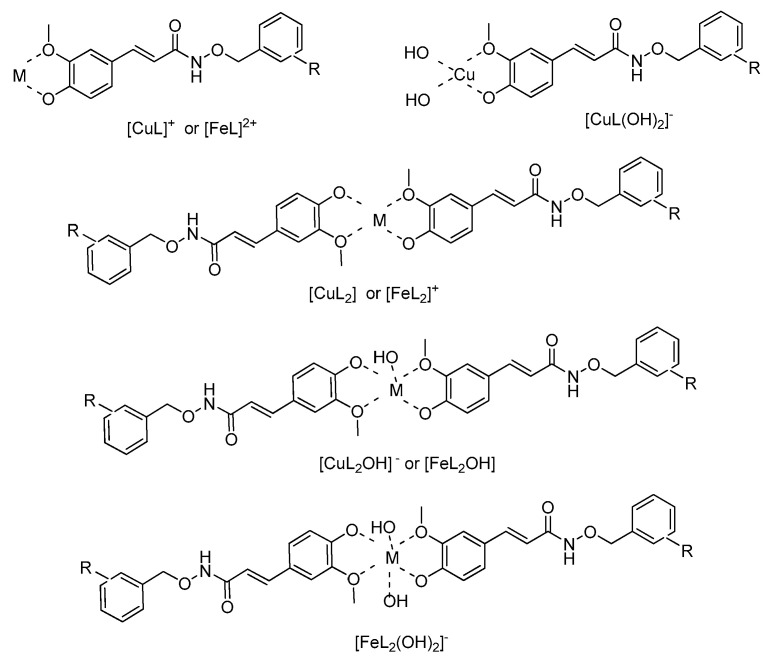
Proposed coordination modes for the complexes of Fe^3+^ and Cu^2+^ with compounds **1a** and **1d**.

The potentiometric curves and the equilibrium models obtained for **FA** seem to point toward the formation of metal complexes involving the carboxylic and the phenolic group for acidic lower pH values, as pointed out in the literature [45,46]. The co-existence of a bidentate *O*-phenol, *O*-methoxy coordination is also believed to be involved, with predominance at higher pH values. In fact, this bidentate *O*-phenol, *O*-methoxy coordination core occurs in the complexation of compounds **1a** and **1d** (see Figure 4) and so it must also compete with the carboxylate group in the M/L systems of **FA**.

In the obtained metal complex models for **FA**, MHL corresponds to the species with the ligand phenolic oxygen atom protonated. ML, ML_2_ and ML_3_ species correspond to complexes involving the completely deprotonated form of the ligand, while MH_-1_L, MH_-2_L, MH_-1_L_2_ and MH_-2_L_2_ are mixed ligand–hydroxide metal complexes.

The stability constants found for the metal complex systems Fe^3+^/**FA** and Cu^2+^/**FA** are similar to those already published [45,46] and small differences between the respective values can be attributed to the different working media and ionic strength.

**Figure 5:**
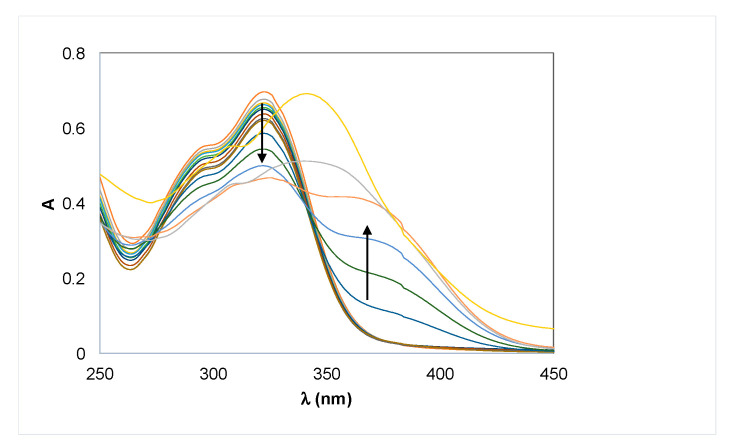
Spectrophotometric absorption spectra of the system Fe^3+^/**1d** 1:3 (2.59 < pH < 10.22) (*C*_L_ = 4.0 × 10^−5^ M, 20% DMSO/water).

Figure 5 presents an illustrative example of the spectral data obtained along the spectrophotometric titration of the Fe^3+^/**1d** system (1:3). 

**Figure 6:**
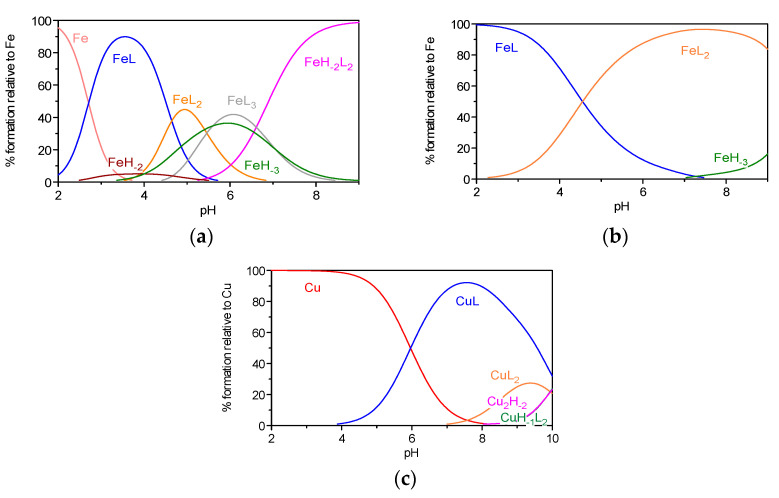
Species distribution curves for the systems: (**a**) Fe^3+^/**FA**, 1:3 (*C***_L_** = 5 × 10^−4^ M); (**b**) Fe^3+^/**1a**, 1:2 (*C***_L_** = 4 × 10^−5^ M); (**c**) Cu^2+^/**1d** 1:2 (*C***_L_** = 4 × 10^−5^ M).

Species distribution curves for some of the 1:2 and 1:3 M/L systems herein studied, at the used experimental conditions, are reported in Figure 6.

From the analysis of Table 2 and Figure 6, **FA** appears as a somewhat weaker iron chelator than **1a** at low pH values, since at pH ca. 2, there is free iron in the Fe^3+^/**FA** system (ca. 90%), while in the presence of **1a**, there is 100% FeL. In fact, as already stated, at low pH, the metal complexation with **FA** seems to occur between the carboxylic group and the phenolic oxygen [28,29]. Moreover, evidence for the formation of FeL_3_ complexes involving **1a** or **1d** was not found from the treatment of the experimental data.

Concerning the Cu^2+^/L systems, the coordination with this metal ion typically occurs at higher pH values than with Fe^3+^, which indicates that **FA**, **1a** and **1d** are stronger chelators for iron than for copper ions.

Comparison of the metal chelating capacity of the studied compounds can be performed by analysis of the respective pM values (at pH 7.4, *C*_L_/*C*_M_ = 10, *C*_M_ = 10^−6^ M) [47]. Both compounds **1a** and **1d** have similar chelating capacities towards iron and copper, which are also analogous to those of **FA**. Thus, these results suggest that in both cases, the metal coordination should involve mostly feroyl phenolic groups, even though the **FA** carboxylic groups may also have some role at the lowest pH values.

### 2.3. In Vitro and In Silico Studies

#### 2.3.1. Inhibition of Aβ_1–42_ Aggregation

The studied compounds (**1a**–**e**) were tested in vitro to evaluate their activity as inhibitors of Aβ_1-42_ peptide aggregation, through the Thioflavin T (ThT) fluorescence assay [48,49]. This method is based on the strong ability of the dye ThT to bind β-amyloid fibrils and oligomers through ionic and hydrophobic interactions, while its interaction with β-amyloid monomers is extremely weak. The intercalation of compounds in the β-sheet structure of amyloid protein may prevent fibrils aggregation and ThT binding, and it is evaluated by fluorimetry. The fluorescence emission measurements were performed after overnight incubation of the self-mediated or Cu^2+^-induced Aβ fibril aggregates with the studied compounds. 

Results are expressed as percentage of aggregation inhibition (Table 3) and tacrine was assayed as a model compound to verify the validity of the method used. All compounds present a good-moderate level of inhibition in both kinds of induced Aβ aggregation. Among the **FA** benzyloxyamide derivatives **1a**–**e**, the chlorine substituent (**1d**, **1e**) seems to slightly decrease the self-aggregation activity of β-amyloid; otherwise, compounds **1a** and **1b** substituted with the -OCH_3_ group, *ortho* and *meta*, and **1c** with the CF_3_ group in the *ortho* position, have higher activities toward self-mediated β-amyloid aggregation. 

All the tested ligands showed an increase in the inhibitory activity for β-amyloid aggregation, when in the presence of the biometal ion Cu^2+^, probably due to their capacity for metal chelation.

#### 2.3.2. In Silico Pharmacokinetic Properties

To predict the drug-likeness of the studied compounds, pharmacokinetic properties were evaluated using in silico tools, namely descriptors provided by the QIKPROP program (v. 2.5) [51]. To estimate compounds’ absorption across biological membranes and their possible toxicity, the following parameters (Table 4) have been calculated: the lipo-hydrophilic character (clog *P*), the blood–brain barrier (BBB) partition coefficient (log BB), the ability to be absorbed through the intestinal tract (Caco-2 cell permeability) and the CNS (Central Nervous System) activity, along with verification of Lipinski’s rule of five. These descriptors are useful to have an idea of the possible formulation for oral use of new compounds as anti-AD drugs. 

From analysis of the results reported in Table 4, which includes compounds **1a**–**e** as well as the parent **FA**, all the compounds are small molecules with low molecular weight (MW) and are aligned with the five points of Lipinski’s rule.

Log BB is calculated dividing the concentration of drug in the brain by the concentration in the blood and it measures the ability of drugs to pass the BBB. All compounds have a log BB value inside the range accepted to be potentially carried to the brain. 

In investigating ADME of novel pharmaceutical molecules, another important step is the prediction of human intestinal permeation by non-active transport, expressed by Caco-2 permeability, as it is usually determined by Caco-2 cell line derived from human colorectal epithelial cancer [52]. The rate of absorption calculated for each compound is high or extremely high as concerns compound **1e**, considering 500 nm/s as the value of reference for great permeability, which represents a good improvement relative to **FA** (87 nm/s). Furthermore, the estimation of CNS activity of studied ligands is not high, although they might pass the BBB. This result can be related to an unlikely cerebral toxicity. 

Overall, the pharmacokinetic parameters depicted in Table 4 were found to be within the acceptable range (see Table 4 footnote).

## 3. Materials and Methods

### 3.1. Chemistry

#### 3.1.1. Materials and Methods

Analytical grade reagents and solvents were purchased from Sigma-Aldrich (St. Louis, MO, USA) and Alfa Aesar (ThermoFisher—Kandel, Germany) and they were used without further purification. Chemical reactions were monitored by Thin Layer Chromatography (TLC) on 0.25 mm aluminum plates, pre-coated with silica gel and containing a fluorescent indicator (Merck Silica Gel 60 F254). The spots on TLC were visualized by a UV lamp (254 nm). Na_2_SO_4_ was used as a dehydrating agent for organic solutions, while their evaporation was carried out under vacuum conditions in a rotating evaporator.

Flash chromatography purifications were carried out in glass columns with silica gel 230–400 mesh (Merck). The characterization of compounds and the assessment of their pureness were performed by the determination of melting points, by NMR and Mass Spectrometry techniques. Melting points (m.p.) were measured with a Leica Galen III microscope. ^1^H and ^13^C NMR spectra were recorded in the proper solvents with a Bruker Ultrashield™ 400 MHz (Fallander, Switzerland), at 25 °C. Chemical shifts (δ) are given in ppm, referring to the signal of the internal standard TMS. Coupling constant values (*J*) are reported in Hz. Signals in NMR spectra are indicated by the following abbreviations: *s* = singlet, *d* = doublet*, m* = multiplet, *dd* = doublet of doublet. Mass spectra (ESI-MS) were obtained on a 500 MS LC Ion Trap mass spectrometer (Varian Inc., Palo Alto, CA, USA) supplied with an ESI ion source. The ^1^H-NMR, ^13^C-NMR and the mass spectra for compounds **1a-e** are available on Supplementary Materials.

#### 3.1.2. General Procedure for the Synthesis of Ferulic Acid Benzyloxyamidic Derivatives (**1a**–**e**)

A solution of commercially available (*E*)-4-hydroxy-3-methoxy cinnamic acid (ferulic acid, FA) (**6**), (1 eq) in anhydrous DMF (2 mL) under inert atmosphere for Argon, was treated with hydroxybenzotriazole (HOBt) (1.2 eq), *N*-methylmorpholine (3 eq), the opportune benzylhydroxylamine hydrochloride **5a**–**e** (3.1 eq) and *N*-(3-dimethylaminopropyl)-*N’*-ethylcarbodiimide hydrochloride (EDCI) (1.4 eq). The resulting mixture was stirred at room temperature (r.t.) for 24 h. Then, the mixture was extracted with AcOEt and washed with H_2_O. The organic phase was dried, filtered, and evaporated to give the crude derivatives that, after purification by flash chromatography (eluent: CHCl_3_, MeOH and NH_3_ in ratio 9:1:0.1, respectively), afforded the corresponding hybrid FA compounds **1a**–**e**.

*(E)-3-(4-hydroxy-3-methoxyphenyl)-N-((2-methoxybenzyl)oxy)acrylamide (**1a**)* Compound **1a** was obtained as a pale yellow solid. Yield: 57%; m.p.: 58 °C. ^1^H-NMR (400 MHz, CD_3_OD-*d_4_*) δ: 7.54–7.50 (d, 1H, *J* = 15.8 Hz, CH=CH); 7.39–7.32 (m, 2H, Ar); 7.11 (s, 1H, Ar) 7.05–6.94 (m, 4H, Ar); 6.81–6.79 (d, 1H, *J* = 8.0 Hz, Ar); 6.25–6.21 (d, 1H, *J* = 15.8 Hz, CH=CH); 4.98 (s, 2H, OCH_2_); 3.88 (s, 3H, OCH_3_); 3.85 (s, 3H, OCH_3_). ^13^C-NMR (100 MHz, CD_3_OD-*d_4_*) δ: 166.9 (1C, C=O); 159.7 (1C, Ar-OCH_3_); 150.2 (1C, Ar-OCH_3_); 149.3 (1C, Ar-OH); 142.9 (1C, CH=CH); 132.2 (1C, Ar); 131.3 (1C, Ar-CH=CH); 128.1, 125.1, 123.3 (3C, Ar); 121.4 (1C, CH=CH), 116.5, 114.9, 111.8 (4C, Ar); 74.0 (OCH_2_); 56.4, 56.0 (2C, OCH_3_). *m/z* ESI-MS: [M + H]^+^ 329.96.
*(E)-3-(4-hydroxy-3-methoxyphenyl)-N-((3-methoxybenzyl)oxy)acrylamide (**1b**)* Compound **1b** was obtained as a solid. Yield: 58.3%; m.p.: 63–65 °C. ^1^H-NMR (400 MHz, CD_3_OD-*d_4_*) δ: 7.54–7.50 (d, 1H, *J* = 16.0 Hz, CH=CH); 7.31-7.25 (m, 1H, Ar); 7.10 (s, 1H, Ar); 7.04–6.99 (m, 3H, Ar); 6.92-6.90 (dd, 2H, *J_1_ =* 8.2 Hz, *J_2_ =* 2.0 Hz, Ar); 6.80–6.78 (d, 1H, *J =* 8.2 Hz, Ar); 6.25-6.21 (d, 1H, *J* = 16.0 Hz, CH=CH); 4.98 (s, 2H, OCH_2_); 3.88 (s, 3H, OCH_3_); 3.81 (s, 3H, OCH_3_). ^13^C-NMR (100 MHz, CD_3_OD-*d_4_*) δ: 166.9 (1C, C=O); 161.3, 150.2 (2C, Ar-OCH_3_); 149.3 (1C, Ar-OH), 143.1 (1C, Ar-CH_2_O); 138.5 (1C, CH=CH); 130.5 (1C, Ar-CH=CH); 128.0, 123.4 (2C, Ar); 122.4 (1C, CH=CH), 116.5, 115.3, 114.8, 111.7 (5C, Ar); 79.1 (1C, OCH_2_); 56.4, 55.7 (2C, OCH_3_). *m/z* ESI-MS: [M + H]^+^ 329.96.
*(E)-3-(4-hydroxy-3-methoxyphenyl)-N-((2-(trifluoromethyl)benzyl)oxy)acrylamide (**1c**)* Compound **1c** was obtained as a solid. Yield: 57%; m.p.: 76–77 °C. ^1^H-NMR (400 MHz, CD_3_OD-*d_4_*) δ: 7.82 (s, 1H, Ar); 7.81–7.75 (d, 1H, *J* = 15.2 Hz, CH=CH); 7.73–7.67 (m, 1H, Ar); 7.58–7.53 (m, 2H, Ar); 7.12 (s, 1H, *J* = 1.5 Hz, Ar); 7.06–7.04 (d, 1H, *J* = 8.0 Hz, Ar); 6.83–6.81 (d, 1H, *J =* 8.0 Hz, Ar); 6.28–6.25 (d, 1H, *J* = 15.2 Hz, CH=CH); 5.15 (s, 2H, OCH_2_); 3.89 (s, 3H, OCH_3_). ^13^C-NMR (100 MHz, CD_3_OD-*d_4_*) δ: 165.7 (1C, C=O); 148.8 (1C, Ar-OCH_3_); 147.9 (1C, Ar-OH); 141.8 (1C, CH=CH); 134.1, 132.0 (2C, Ar); 131.1 (1C, Ar-CH=CH); 128.5 (1C, Ar); 126.6 (1C, Ar-CF_3_); 125.7 (1C, Ar); 125.5 (1C, Ar); 123.0 (1C, CF_3_); 122.0 (1C, Ar); 115.1 (1C, CH=CH); 113.2, 110.3 (2C, Ar); 73.7 (1C, OCH_2_); 55.0 (1C, OCH_3_). *m/z* ESI-MS: [M + H]^+^ 368.08.
*(E)-N-((3-chlorobenzyl)oxy)-3-(4-hydroxy-3-methoxyphenyl)acrylamide (**1d**)* Compound **1d** was obtained as a yellow solid. Yield: 64%; m.p.: 47–48 °C. ^1^H-NMR (400 MHz, CD_3_OD-*d_4_*) δ: 7.54–7.50 (d, 1H, *J* = 15.0 Hz, CH=CH); 7.50 (s, 1H, Ar); 7.38–7.35 (m, 3H, Ar); 7.11–7.10 (d, 1H, *J* = 1.9 Hz, Ar); 7.04-7.01 (dd, 1H, *J_1_ =* 8.4 Hz, *J_2_* = 1.9 Hz, Ar); 6.80–6.78 (d, 1H, *J* = 8.4 Hz, Ar); 6.25-6.21 (d, 1H, *J* = 15.0 Hz, CH=CH); 4.90 (s, 2H, OCH_2_); 3.87 (s, 3H, OCH_3_). ^13^C-NMR (100 MHz, CD_3_OD-*d_4_*) δ: 167.0 (1C, C=O); 150.1 (1C, Ar-OCH_3_); 149.2 (1C, Ar-OH) 143.1 (1C, Ar-CH_2_O); 139.3 (1C, CH=CH); 135.3 (1C, Ar-Cl); 130.9 (1C, Ar-CH=CH); 130.0, 129.5, 128.4, 127.8, 123.3 (5C, Ar); 116.4 (1C, CH=CH); 114.5, 111.5 (2C, Ar); 78.2 (1C, OCH_2_); 56.3 (1C, OCH_3_). *m/z* ESI-MS: [M + H]^+^ 334.04.
*(E)-N-((2,4-dichlorobenzyl)oxy)-3-(4-hydroxy-3-methoxyphenyl)acrylamide (**1e**)* Compound **1e** was obtained as a pale yellow solid. Yield: 69%; m.p.: 164.3-166.5 °C. ^1^H-NMR (400 MHz, CD_3_OD-*d_4_*) δ: 7.57 (s, 2H, Ar); 7.55–7.51 (d, 1H, *J* = 15.0 Hz, CH=CH); 7.39–7.37 (dd, 1H, *J_1_ =* 8.2 Hz, *J_2_ =* 1.8 Hz, Ar); 7.11–7.10 (d, 1H, *J =* 1.8 Hz, Ar); 7.04-7.02 (dd, 1H, *J_1_ =* 8.2 Hz, *J_2_ =* 1.8 Hz, Ar); 6.81–6.79 (d, 1H, *J* = 8.2 Hz, Ar); 6.24–6.20 (d, 1H, *J* = 15.0 Hz, CH=CH); 5.04 (s, 2H, OCH_2_); 3.88 (s, 3H, OCH_3_). ^13^C-NMR (100 MHz, CD_3_OD-*d_4_*) δ: 167.2 (1C, C=O); 150.3 (1C, Ar-OCH_3_); 149.3 (1C, Ar-OH) 143.3 (1C, CH=CH); 136.4 (1C, Ar-CH_2_O); 133.7 (2C, Ar-Cl); 130.3 (1C, Ar-CH=CH); 128.4, 128.0, 123.5 (3C, Ar); 123.4 (1C, CH=CH); 116.5, 114.6, 111.7 (3C, Ar); 75.3 (1C, OCH_2_); 56.4 (1C, OCH_3_). *m/z* ESI-MS: [M + H]^+^ 367.94.

### 3.2. Physicochemical and Biological Properties 

#### 3.2.1. Materials and Methods

For the physicochemical and biological studies, analytical grade reagents and solvents were supplied by Sigma-Aldrich (St. Louis, MO, USA), Fluka (Buchs, Switzerland) and Acros (Geel, Belgium). They were used as received without any purification. The antioxidant activity assay was performed in a quartz cell of 1 cm path length with a Perkin-Elmer Lambda 35 UV–Vis spectrophotometer equipped with a temperature programmer PTP1+1 Peltier System (*T* = 25.0 ± 0.1 °C). 

As concerns the metal chelation studies, potassium hydrogen phthalate (C_8_H_5_KO_4_, p.a.) was acquired from BDH, whereas dimethylsulfoxide (DMSO, dried, p.a.) and potassium chloride (KCl, p.a.) were supplied by Sigma-Aldrich. Aqueous solutions of metal ions were prepared starting from 1000 ppm standards (Titrisol, Sigma-Aldrich), to obtain solutions FeCl_3_ 0.018 M and CuCl_2_ 0.015 M. The metal content of solutions was evaluated by atomic absorption. Iron stock solution was prepared with an excess of acid chloride to avoid hydrolysis and its concentration in HCl was determined by the usual standard-addition method using 0.1M HCl (Titrisol). The 0.1M HCl solution, used in calibration of the glass electrode and in spectrophotometric titrations, was prepared from a Titrisol ampoule. The titrant was prepared from carbonate free commercial concentrate (Titrisol, KOH 0.1 M ampoules). The KOH solution was standardized by titration with a solution of potassium hydrogen phthalate using Gran’s method and it was rejected when the percentage of carbonate was greater than 0.5% of the total amount of base. The potentiometric studies were performed with an automated potentiometric apparatus controlled by PASAT program and containing a Crison micropH 2002 millivoltmeter, a Crison microBu 2031 burette and a Haake thermostatic bath (*T* = 25.0 ± 0.1 °C). The spectrophotometric titrations were carried out with a Perkin-Elmer Lambda 35 spectrophotometer.

The inhibition of β-amyloid (Aβ) aggregation was studied through the Th-T fluorescence assay, which was performed using a Varian Cary Eclipse fluorimeter at the following wavelengths: excitation (446 nm) and emission (485 nm). Amyloid-β peptide (1–42) was purchased from GeneCust as lyophilized powder stored at −20 °C. 

#### 3.2.2. Antioxidant Activity

Compounds were evaluated for their radical scavenging activity by the Blois method using DPPH (2,2-diphenyl-1-picrylhydrazyl), a stable free-radical. It absorbs in methanol solution at 517–520 nm and it is a scavenger for other radicals converting itself in the reduced form DPPHH. The assay consists of preparing solutions of increasing concentration of each compound starting from a stock solution (2–3 × 10^−4^ M), adding to them 2.5 mL DPPH (0.002% in MeOH, 50–100 µM) and the necessary volume of MeOH to reach the final volume 3.5 mL for each sample. The control sample was made up of DPPH and MeOH. Solutions were protected from light at r.t. for 30 min and then absorbance was measured at 517 nm with a Perkin Elmer Lambda 35 UV–Vis spectrophotometer, using methanolic solution as the blank in the other cell. All the studied compounds have good solubility in MeOH, so their solutions could be prepared. The assay was repeated three times for all compounds and the antioxidant activity, expressed as EC_50_, was calculated by Equation (1).

%AA = ((A_DPPH_ − A_sample_)/A_DPPH_) × 100, (1)

Results correspond to the % of antioxidant activity related to ligand concentration and are expressed by the average EC_50_ values of the three assays, that is the concentration of substrate which causes 50% loss of DPPH activity. 

#### 3.2.3. Metal Chelation: Potentiometric and Spectrophotometric Studies

Titrations of selected compounds (**FA**, **1a**, **1d**) were performed in a 20% *w/w* DMSO/H_2_O medium, at *T* = 25.0 ± 0.1 °C and ionic strength (*I*) 0.1 M KCl, by using 0.1 M KOH as titrant. Both glass and Ag/AgCl reference electrodes were previously conditioned in different DMSO/H_2_O mixtures of increasing DMSO % concentration and the response of the glass electrode was evaluated by strong acid–strong base (HCl/KOH) calibrations with the determination of the Nernst parameters by Gran’s method [53]. The measurements were performed in a final volume of 30.00 mL and the ligand concentrations (*C*_L_) were 5–6.7 × 10^−4^ M for the potentiometric studies and 4 × 10^−5^ M for the spectrophotometric titrations, under different *C*_M_/*C*_L_ (M = Fe, Cu) ratios: 0:1 (L), 1:1, 1:2 and 1:3. The spectrophotometric measurements were carried out in a 250–450 nm wavelength range at pH ca. 2.5–11.8. Under the stated experimental conditions, the p*K*_w_ value (14.27) was determined and subsequently used in the computations. The stepwise protonation constants of the ligands, *K_i_* = [H_i_L]/[H_i-1_L][H], and the overall metal complex stability constants, β (M_m_H_h_L_l_) = [M_m_H_h_L_l_]/[M]^m^[H]^h^[L]^l^, were calculated by fitting the pH-potentiometric and spectrophotometric data with, respectively, the Hyperquad 2008 [41] and Psequad [42] programs. The metal hydrolysis model was determined under the defined experimental conditions (*I* = 0.1 M KCl, 20% w/w DMSO/H_2_O, *T* = 25.0 ± 0.1 °C) and the following values of stability constants were included in the fitting of experimental data towards the equilibrium models related to the Fe^3+^/L and Cu^2+^/L systems: log *β* (FeH_-2_) = −6.78, log β (FeH_-3_) = −10.78; log β (Cu_2_H_-2_) = −9.94. The species distribution curves were obtained with the Hyss program [41].

#### 3.2.4. Inhibition of Self and Cu^2+^-Mediated Aβ_1–42_ Aggregation

The method used is based on thioflavin T (ThT) fluorescence emission, which depends on the interaction of this dye with A*β* fibrils [49]. The assay is performed with the A*β*_1–42_ peptide sample, which was previously prepared by solubilization of its lyophilized powder in 1,1,1,3,3,3–hexafluoro-2-propanol (HFIP), an organic solvent useful to solubilize and monomerize the β-sheets protein aggregates. After 24 h, the solution was divided into Eppendorf tubes kept in ice, and then, HFIP was left to evaporate overnight (*T* = 25 °C). The resultant films were stored in the freezer. To perform the assay, each film of A*β* was re-dissolved in a solution of 69.5 µL CH_3_CN/Na_2_CO_3_ (300 µM)/NaOH (250 mM) in ratio 48.3:48.3:3.4 µL, and 392.5 µL of phosphate buffer 0.215 M (pH = 8) was added to the resulting alkaline solution to obtain a final concentration of 40 μM. Compounds were dissolved in MeOH (1 mg/mL) and diluted with phosphate buffer to reach a ligand stock solution of 480 μM. Two different types of experiments were prepared in a final volume of 60 μL: control ligand assays (Aβ_1–42_ aggregation in the absence of inhibitor) and ligand assays (effect of ligand on Aβ_1–42_ aggregation). For each experiment, a blank sample without Aβ_1–42_ was used to monitor the effect of the compounds in fluorescence. To study the inhibition of aggregation in the presence of Cu^2+^, which is known to promote it, an intermediate stock solution of CuCl_2_ in phosphate buffer (240 µM) was prepared. Then, it was aliquoted to obtain a final concentration of 40 µM in the samples. All samples were incubated in a water bath for 24 h at 37 °C. Then, 180 µL of ThT (5 µM) in glycine-NaOH buffer (50 mM, pH = 8.5) is added to each solution and 200 µL of it is placed in a 96-well plate (BD Falcon) to be read with the fluorimeter. After 5 min of incubation with ThT, fluorescence was measured at 446 nm (ʎ excitation) and 485 nm (ʎ emission).

#### 3.2.5. In Silico ADME Properties

The drug-likeness of all compounds was investigated by in silico calculations using the software QIKPROP (version 2.5) provided by MAESTRO [51]. The following pharmacokinetic descriptors or ADME (absorption, distribution, metabolism and excretion) properties were calculated, namely, to predict: the lipophilicity (clog *P*), the blood–brain barrier partition coefficient (log BB), the ability to be absorbed through the intestinal tract (Caco-2 cell permeability), the CNS activity, along with the verification of Lipinski’s rule of five. The prediction of those parameters gives us an idea of the ADME profile of the new molecules as possible drugs for oral use and of their absorption in the CNS.

## 4. Conclusions

The absence of a cure for the severe and complex Alzheimer’s disease (AD) is the main rationale for the work described herein, focused on the development and study of a new set of multifunctional ferulic acid (FA) derivatives as potential anti-AD agents. In particular, a set of hybrids enclosing the ferulic acid scaffold and benzyloxyamines with different substituent groups were synthesized and evaluated for their physicochemical and biological properties. These compounds demonstrated good chelating capacity towards redox-active metal ions (Cu^2+^ and Fe^3+^) and good radical scavenging capacity, both these properties being conferred by the FA moiety. They also evidenced moderate/good capacity for inhibition of self-induced beta-amyloid (Aβ) aggregation, which was considerably improved in the case of Cu-induced aggregation, attributable to their Cu-chelation ability. Finally, their in silico predicted ADME properties are in the range of known oral drugs and also satisfied Lipinski’s rule of five, indicating good absorption and, hence, good bioavailability. Thus, these compounds appear with promising lead structure for further developments as anti-Alzheimer agents.

## Figures and Tables

**Table 1:** Antioxidant results based on radical scavenging activity using the DPPH method.

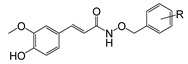 Compound	R	Radical Scavenging Activity ^a^ (EC_50,_ μM)
**1a**	2-OCH_3_	34 ± 1
**1b**	3-OCH_3_	40 ± 2
**1c**	2-CF_3_	34 ± 1
**1d**	3-Cl	33 ± 1
**1e**	2,4-Cl	32 ± 3
**Ferulic** **acid**	-	36 ± 2
**Ascorbic acid**	-	25 ± 1 [40]

^a^ Mean ± SD of 3 independent experiments for 50% antioxidant activity.

**Table 2:** Stepwise protonation constants of **FA**, **1a**, and **1d** as well as global formation constants ^a^ of their Fe^3+^, Cu^2+^ complexes and corresponding pM ^b^ values. (*T* = 25.0 ± 0.1 °C, *I* = 0.1 M KCl, 20% *w/w* DMSO/water).

Compound	M_m_H_h_L_l_ (mhl)	log *K*_i_	log β	
			(Fe_m_H_h_L_l_) ^a^	(Cu_m_H_h_L_l_) ^a^
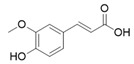 **FA**	(011) (021) (111) (101) (1–11) (102) (1–12) (1–22) (103) **pM**	9.43(2) ^c^ 4.83(4) ^c^	- 12.15(6) ^c^ - 20.31(6) ^c^ - 7.81(8) ^c^ 26.70(8) ^c^ **17.4**	13.30(4) ^c^ 6.52(8) ^c^ - 11.14(6) ^c^ 1.90(8) ^c^ - - **6.2**
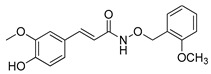 **1a**	(011) (101) (1–21) (102) (1–12) **pM**	8.75(2) ^d^	16.49(3) ^d^ - 23.69(4) ^d^ - **17.6**	6.27(7) ^d^ −13.16(7) ^d^ - 1.12(5) ^d^ **6.3**
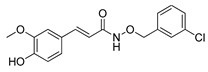 **1d**	(011) (101) (102) (1–12) (1–22) **pM**	8.93(3) ^d^	15.42(7) ^d^ 25.43(5) ^d^ - 8.50(5) **18.2**	7.49(7) ^d^ 12.14(7) ^d^ 2.20(8) ^d^ **-** **7.0**

^a^ β (M_m_H_h_L_l_) = [M_m_H_h_L_l_]/[M]^m^[H]^h^[L]^l^; ^b^ pM = −log [M] at pH 7.4 (*C*_L_/*C*_M_ = 10, *C*_M_ = 10^−6^ M); ^c^ pH-potentiometric data; ^d^ UV–Vis spectrophotometric data.

**Table 3:** Inhibitory capacity of the compounds for Aβ_1–42_ aggregation *.

Compound	R	Inhibition of Aβ_1–42_ Aggregation (%)	
		Self-Aβ Aggr.	Cu-Induced Aβ Aggr.
**1a**	2-OCH_3_	42.6	79.6
**1b**	3-OCH_3_	52.8	69.5
**1c**	2-CF3	51.0	68.8
**1d**	3-Cl	34.1	76.1
**1e**	2,4-Cl	28.3	77.0
**Tacrine**	-	21.5, 22.8 [50]	-

* Thioflavin-T fluorescence method in the presence of compounds (80 μM) with or without copper (40 μM). The values are the mean of two independent measurements in duplicate for Aβ [49].

**Table 4:** Physicochemical descriptors and ADME properties of tested compound **1(a**–**e)** calculated by QIKPROP v. 2.5 [51].

Compound	MW ^a^	clog *P* ^b^	Log BB ^c^	P_Caco-2_^d^	Oral Absorp. ^e^	CNS Act. ^f^	Violations Rule of 5 ^g^
**1a**	329.35	−2.006	−1.206	579	84	--	0
**1b**	329.35	−2.016	−1.196	579	84	--	0
**1c**	367.32	−0.905	−1.073	388	77	--	0
**1d**	333.45	−1.073	−0.976	579	84	-	0
**1e**	367.9	−0.516	−0.752	732	85	-	0
**FA**	194.2	1.447	−1.03	87	61	--	0

^a^ (Acceptable <500); ^b^ Predicted octanol/water partition coefficient log P (acceptable range −2.0 to 6.5); ^c^ Predicted BBB permeability (acceptable range −3 to 1.2); ^d^ Predicted Caco-2 cell permeability in nm/s (acceptable range: <25 is poor and >500 is great); ^e^ Percentage of human oral absorption (acceptable range: <25% is poor and >80% is high); ^f^ qualitative CNS activity parameter from (--) inactive, (++) active; ^g^ Number of violations of Lipinski’s rule of five. The rules are MW < 500, clog P_o/w_ < 5, donor HB ≤ 5, acceptor HB ≤ 10. Compounds that satisfy these rules are considered drug-like (the “five” refers to the limits, which are multiples of 5).

## Data Availability

“MDPI Research Data Policies” at https://www.mdpi.com/ethics, accessed on 1 October 2021.

## Supplementary Materials

The ^1^H-NMR, ^13^C-NMR and the mass spectra for compounds **1a**–**e** are available online.

## Sample Availability

Samples of the compounds **1a**–**e** are available from the authors.

## References

[1] Alzheimer’s Association (2019). 2019 Alzheimer’s Disease Facts and Figures. Alzheimer Dement..

[2] Alzheimer’s Disease International World Alzheimer Report 2019. https://www.alz.co.uk/research/WorldAlzheimerReport2019.pdf.

[3] Ittner L.M., Götz J. (2011). Amyloid-β and Tau—A toxic pas de deux in Alzheimer’s disease. Nat. Rev. Neurosci..

[4] Frontiers in Clinical Drug Research—Alzheimer Disorders. https://www.eurekaselect.com/170302/volume/8.

[5] Canady V.A. (2021). FDA Approves First Drug Therapy for Alzheimer’s in 18 Years. Ment. Health Wkly..

[6] Liao L., Cheng D., Wang J., Duong D.M., Losik T.G., Gearing M., Rees H.D., Lah J.J., Levey A.I., Peng J. (2004). Proteomic Characterization of Postmortem Amyloid Plaques Isolated by Laser Capture Microdissection. J. Biol. Chem..

[7] Ciccone L., Shi C., di Lorenzo D., Van Baelen A.-C., Tonali N. (2020). The Positive Side of the Alzheimer’s Disease Amyloid Cross-Interactions: The Case of the Aβ 1-42 Peptide with Tau, TTR, CysC, and ApoA1. Molecules.

[8] Ciccone L., Fruchart-Gaillard C., Mourier G., Savko M., Nencetti S., Orlandini E., Servent D., Stura E.A., Shepard W. (2018). Copper Mediated Amyloid- β Binding to Transthyretin. Sci. Rep..

[9] An S., Fu L. (2018). Small-Molecule PROTACs: An Emerging and Promising Approach for the Development of Targeted Therapy Drugs. EBioMedicine.

[10] Xi M., Chen Y., Yang H., Xu H., Du K., Wu C., Xu Y., Deng L., Luo X., Yu L. (2019). Small Molecule PROTACs in Targeted Therapy: An Emerging Strategy to Induce Protein Degradation. Eur. J. Med. Chem..

[11] Tonali N., Nencetti S., Orlandini E., Ciccone L. (2021). Application of PROTAC Strategy to TTR-Aβ Protein-Protein Interaction for the Development of Alzheimer’s Disease Drugs. Neural Regen. Res..

[12] Raj K., Kaur P., Gupta G.D., Singh S. (2021). Metals Associated Neurodegeneration in Parkinson’s Disease: Insight to Physiological, Pathological Mechanisms and Management. Neurosci. Lett..

[13] Kenche V.B., Barnham K.J. (2011). Alzheimer’s Disease & Metals: Therapeutic Opportunities. Br. J. Pharmacol..

[14] Ciccone L., Policar C., Stura E.A., Shepard W. (2016). Human TTR Conformation Altered by Rhenium Tris-Carbonyl Derivatives. J. Struct. Biol..

[15] Yang G., Liu H., Ma D.-L., Leung C.-H. (2019). Rebalancing Metal Dyshomeostasis for Alzheimer’s Disease Therapy. J. Biol. Inorg. Chem..

[16] Shcherbatykh I., Carpenter D.O. (2007). The Role of Metals in the Etiology of Alzheimer’s Disease. J. Alzheimer’s Dis. JAD.

[17] Ciccone L., Tonali N., Shepard W., Nencetti S., Orlandini E. (2021). Physiological Metals Can Induce Conformational Changes in Transthyretin Structure: Neuroprotection or Misfolding Induction?. Crystals.

[18] Cicero C.E., Mostile G., Vasta R., Rapisarda V., Signorelli S.S., Ferrante M., Zappia M., Nicoletti A. (2017). Metals and Neurodegenerative Diseases. A Systematic Review. Environ. Res..

[19] Nam G., Lim M.H. (2019). Intertwined Pathologies of Amyloid-β and Metal Ions in Alzheimer’s Disease: Metal–Amyloid-β. Chem. Lett..

[20] Kepp K.P., Squitti R. (2019). Copper Imbalance in Alzheimer’s Disease: Convergence of the Chemistry and the Clinic. Coord. Chem. Rev..

[21] Himes R.A., Park G.Y., Siluvai G.S., Blackburn N.J., Karlin K.D. (2008). Structural Studies of Copper(I) Complexes of Amyloid-β Peptide Fragments: Formation of Two-Coordinate Bis(Histidine) Complexes. Angew. Chem..

[22] Santos M.A., Chand K., Chaves S. (2016). Recent Progress in Multifunctional Metal Chelators as Potential Drugs for Alzheimer’s Disease. Coord. Chem. Rev..

[23] Chaves S., Várnagy K., Santos M.A. (2021). Recent Multi-Target Approaches on the Development of Anti- Alzheimer’s Agents Integrating Metal Chelation Activity. Curr. Med. Chem..

[24] Barbosa M., Valentão P., Andrade P.B. (2020). Polyphenols from Brown Seaweeds (*Ochrophyta**, Phaeophyceae*): Phlorotannins in the Pursuit of Natural Alternatives to Tackle Neurodegeneration. Mar. Drugs.

[25] Pereira L., Valado A. (2021). The Seaweed Diet in Prevention and Treatment of the Neurodegenerative Diseases. Mar. Drugs.

[26] Choi B.W., Lee H.S., Shin H.-C., Lee B.H. (2015). Multifunctional Activity of Polyphenolic Compounds Associated with a Potential for Alzheimer’s Disease Therapy from Ecklonia Cava. Phytother. Res..

[27] Polsinelli I., Nencetti S., Shepard W., Ciccone L., Orlandini E., Stura E.A. (2016). A New Crystal Form of Human Transthyretin Obtained with a Curcumin Derived Ligand. J. Struct. Biol..

[28] Airoldi C., La Ferla B., D’Orazio G., Ciaramelli C., Palmioli A. (2018). Flavonoids in the Treatment of Alzheimer’s and Other Neurodegenerative Diseases. Curr. Med. Chem..

[29] Andrade S., Ramalho M.J., Loureiro J.A., Pereira M.D.C. (2019). Natural Compounds for Alzheimer’s Disease Therapy: A Systematic Review of Preclinical and Clinical Studies. Int. J. Mol. Sci..

[30] Martins M., Silva R., Pinto M.M.M., Sousa E. (2020). Marine Natural Products, Multitarget Therapy and Repurposed Agents in Alzheimer’s Disease. Pharmaceuticals.

[31] Ciccone L., Vandooren J., Nencetti S., Orlandini E. (2021). Natural Marine and Terrestrial Compounds as Modulators of Matrix Metalloproteinases-2 (MMP-2) and MMP-9 in Alzheimer’s Disease. Pharmaceuticals.

[32] Sgarbossa A., Giacomazza D., Di Carlo M. (2015). Ferulic Acid: A Hope for Alzheimer’s Disease Therapy from Plants. Nutrients.

[33] Singh Y.P., Rai H., Singh G., Singh G.K., Mishra S., Kumar S., Srikrishna S., Modi G. (2021). A Review on Ferulic Acid and Analogs Based Scaffolds for the Management of Alzheimer’s Disease. Eur. J. Med. Chem..

[34] Balsamo A., Belfiore M., Macchia M., Martini C., Nencetti S., Orlandini E., Rossello A. (1994). Synthesis and Aldose Reductase Inhibitory Activity of *N*-(Arylsulfonyl)- and *N*-(Aroyl)-*N*-(Arylmethyloxy)Glycines. Eur. J. Med. Chem..

[35] Gentili D., Macchia M., Menchini E., Nencetti S., Orlandini E., Rossello A., Broccali G., Limonta D. (1999). Synthesis and Antimicrobial Properties of Cephalosporin Derivatives Substituted on the C(7) Nitrogen with Arylmethyloxyimino or Arylmethyloxyamino Alkanoyl Groups. Il Farm..

[36] Ciccone L., Nencetti S., Camodeca C., Ortore G., Cuffaro D., Socci S., Orlandini E. (2021). Synthesis and Evaluation of Monoaryl Derivatives as Transthyretin Fibril Formation Inhibitors. Pharm. Chem. J..

[37] Borges F., Lima J.L.F.C., Pinto I., Reis S., Siquet C. (2003). Application of a Potentiometric System with Data-Analysis Computer Programs to the Quantification of Metal-Chelating Activity of Two Natural Antioxidants: Caffeic Acid and Ferulic Acid. Helv. Chim. Acta.

[38] Šebestík J., Marques S.M., Falé P.L., Santos S., Arduíno D.M., Cardoso S.M., Oliveira C.R., Serralheiro M.L.M., Santos M.A. (2011). Bifunctional Phenolic-Choline Conjugates as Anti-Oxidants and Acetylcholinesterase Inhibitors. J. Enzym. Inhib. Med. Chem..

[39] David J., Barreiros A., David J. (2004). Antioxidant Phenylpropanoid Esters of Triterpenes from *Dioclea Lasiophylla*. Pharm. Biol..

[40] Naksuriya O., Okonogi S. (2015). Comparison and Combination Effects on Antioxidant Power of Curcumin with Gallic Acid, Ascorbic Acid, and Xanthone. Drug Discov. Ther..

[41] Gans P., Sabatini A., Vacca A. (1996). Investigation of Equilibria in Solution. Determination of Equilibrium Constants with the HYPERQUAD Suite of Programs. Talanta.

[42] Zékány L., Nagypál I., Peintler G. (2001). PSEQUAD, Version 5.01.

[43] Beneduci A., Furia E., Russo N., Marino T. (2017). Complexation Behaviour of Caffeic, Ferulic and p-Coumaric Acids towards Aluminium Cations: A Combined Experimental and Theoretical Approach. New J. Chem..

[44] Casolaro M., Anselmi C., Picciocchi G. (2005). The Protonation Thermodynamics of Ferulic Acid/γ-Cyclodextrin Inclusion Compounds. Thermochim. Acta.

[45] Angkawijaya A.E., Fazary A.E., Hernowo E., Taha M., Ju Y.-H. (2011). Iron(III), Chromium(III), and Copper(II) Complexes of l -Norvaline and Ferulic Acid. J. Chem. Eng. Data.

[46] Khvan A.M., Kristallovich E.L., Abduazimov K.A. (2001). Complexation of Caffeic and Ferulic Acids by Transition-Metal Ions. Chem. Nat. Compd..

[47] Raymond K.N., Carrano C.J. (1979). Coordination Chemistry and Microbial Iron Transport. Acc. Chem. Res..

[48] Biancalana M., Koide S. (2010). Molecular Mechanism of Thioflavin-T Binding to Amyloid Fibrils. Biochim. Et Biophys. Acta (BBA) Proteins Proteom..

[49] Piemontese L., Tomás D., Hiremathad A., Capriati V., Candeias E., Cardoso S.M., Chaves S., Santos M.A. (2018). Donepezil Structure-Based Hybrids as Potential Multifunctional Anti-Alzheimer’s Drug Candidates. J. Enzym. Inhib. Med. Chem..

[50] Chao X., He X., Yang Y., Zhou X., Jin M., Liu S., Cheng Z., Liu P., Wang Y., Yu J. (2012). Design, Synthesis and Pharmacological Evaluation of Novel Tacrine–Caffeic Acid Hybrids as Multi-Targeted Compounds against Alzheimer’s Disease. Bioorg. Med. Chem. Lett..

[51] (2005). QikProp, Version 2.5.

[52] Artursson P., Palm K., Luthman K. (2012). Caco-2 Monolayers in Experimental and Theoretical Predictions of Drug Transport. Adv. Drug Deliv. Rev..

[53] Rossotti F.J.C., Rossotti H. (1965). Potentiometric Titrations Using Gran Plots: A Textbook Omission. J. Chem. Educ..

