# Supplementary material for: Multifunctional Small Molecules as Potential Anti-Alzheimer’s Disease Agents
Source: Molecules. 2021 Oct 3;26(19):6015. doi: 10.3390/molecules26196015 (PMC8512147; doi:10.3390/molecules26196015)

Supplementary Materials

Multifunctional small molecules as potential anti-Alzheimer's disease agents

Beatrice Bargagna [1,3 $], Lidia Ciccone [2, $], Susanna Nencetti [2], Maria Amélia Santos [3,*], Sílvia Chaves [3], Caterina Camodeca [2] and Elisabetta Orlandini [1,4*]

[1] *Department of Earth Sciences, University of Pisa, Via Santa Maria 53-55, 56100, Pisa, Italy*; beatrice.bargagna@dst.unipi.it (B.B.);
[2] *Department of Pharmacy, University of Pisa, Via Bonanno 6, 56126, Pisa, Italy.*; lidia.ciccone@unipi.it (L.C.); susanna.nencetti@unipi.it (S.N.); caterina.camodeca@unipi.it (C.C.);
[3] *Centro de Química Estrutural and Departamento de Engenharia Química, Instituto Superior Técnico, Universidade de Lisboa, Av. Rovisco Pais, 1049-001 Lisboa, Portugal.*; masantos@tecnico.ulisboa.pt (M.A.S.); silvia.chaves@tecnico.ulisboa.pt (S.C.);
[4] *Research Center "E. Piaggio", University of Pisa, Pisa, 56122, Italy*; elisabetta.orlandini@unipi.it (E.O.);

$ *these authors contributed equally to the manuscript*

* Correspondence: elisabetta.orlandini@unipi.it E.O.; masantos@tecnico.ulisboa.pt M.A.S.

Table of contents

Compounds 1a-e. ^{1}H NMR and ^{13}C NMR. 1-5

Mass spectra compounds 1a-e. 6-8

^{1}H and ^{13}C NMR (MeOD) spectra of compound **1a**.

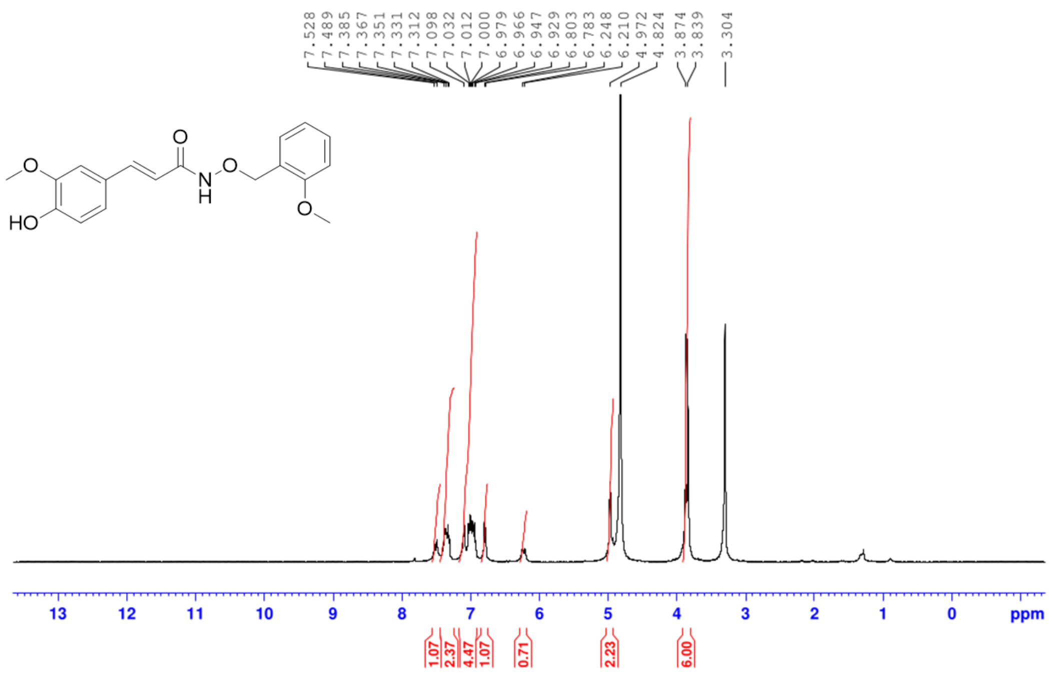

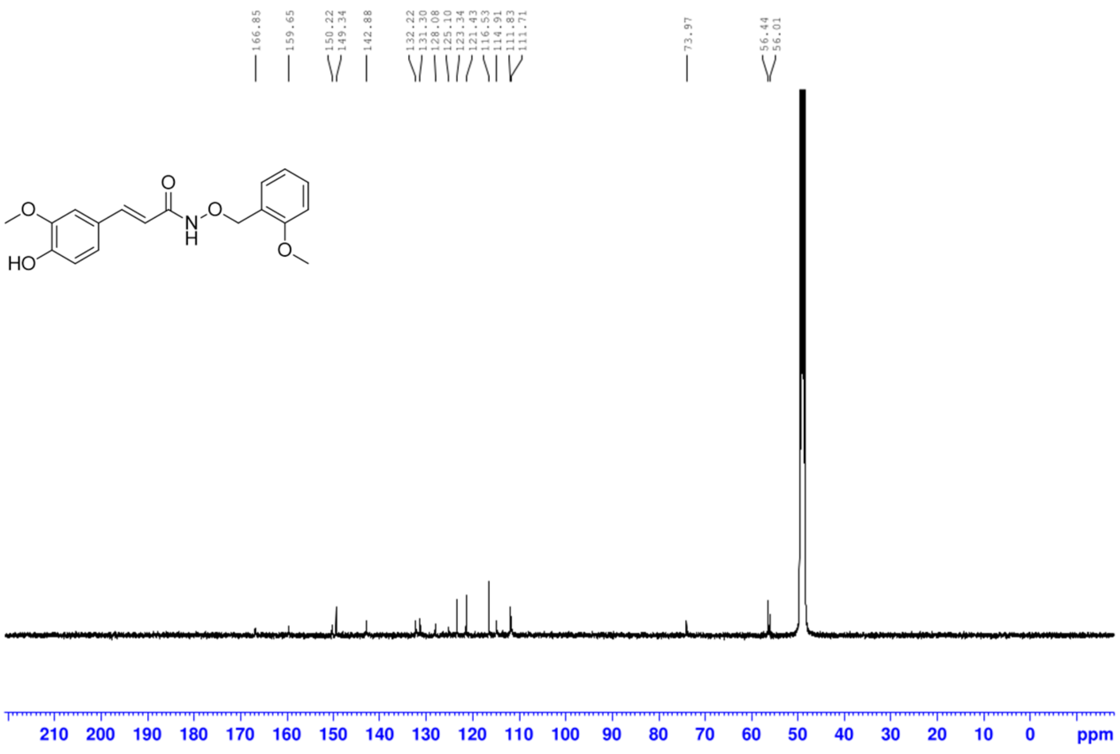

^{1}H and ^{13}C NMR (MeOD) spectra of compound **1b**.

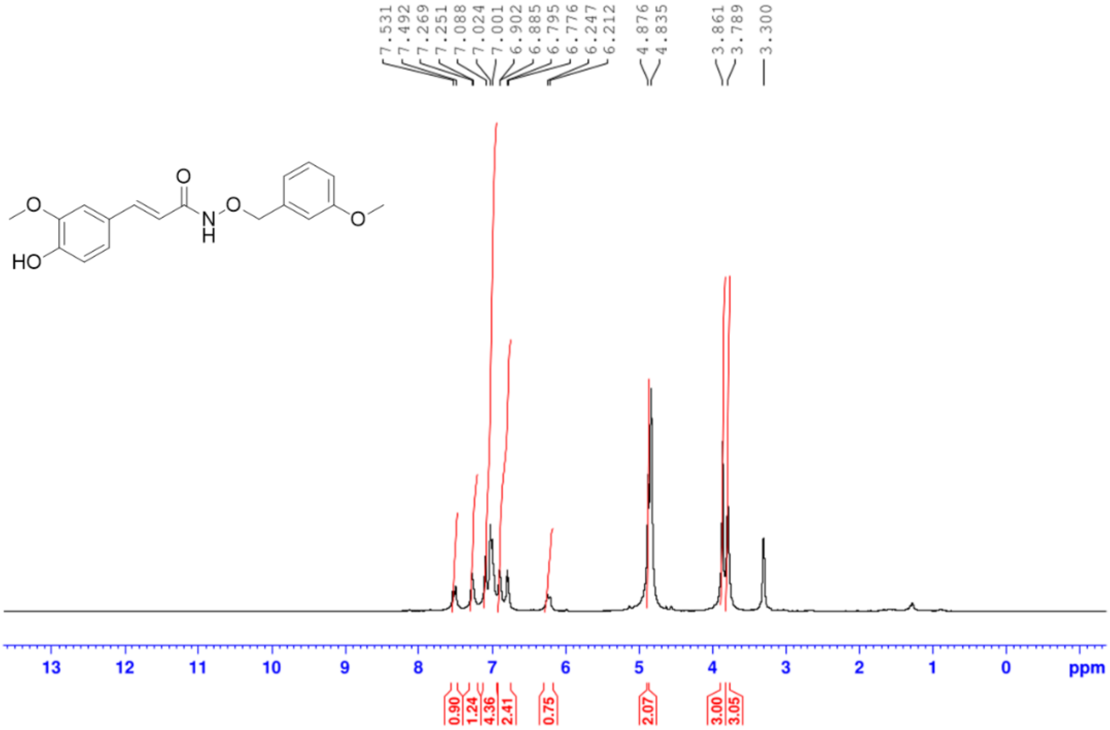

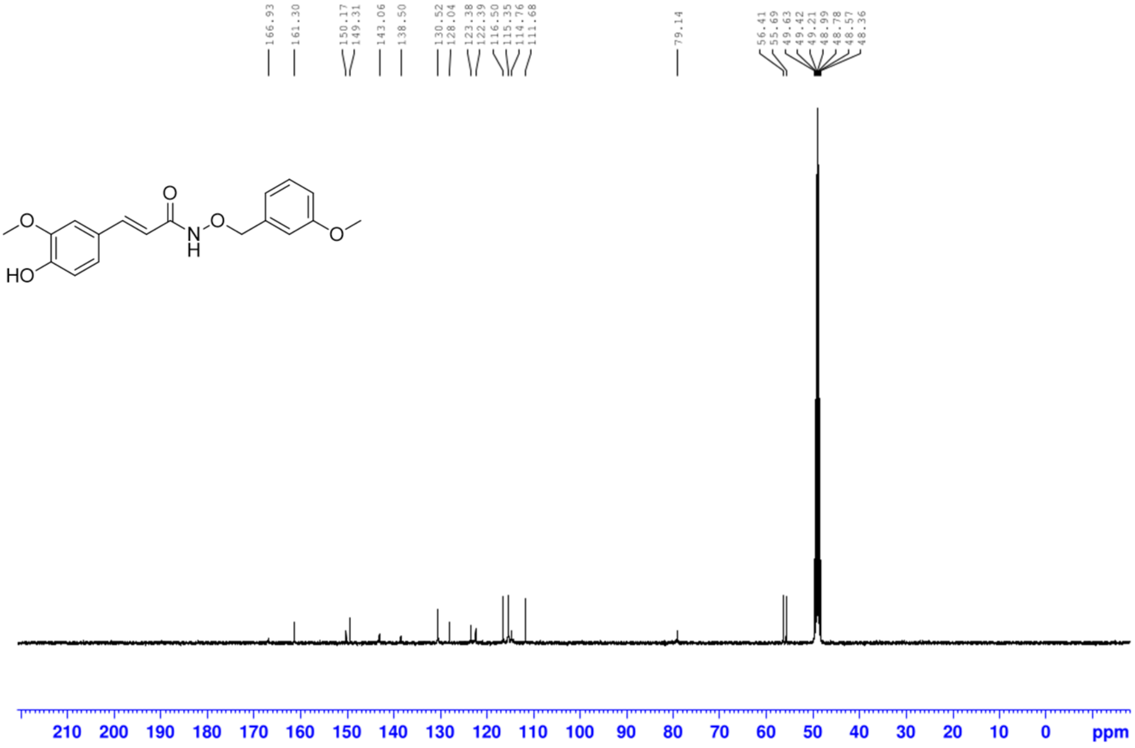

^{1}H and ^{13}C NMR (MeOD) spectra of compound **1c**.

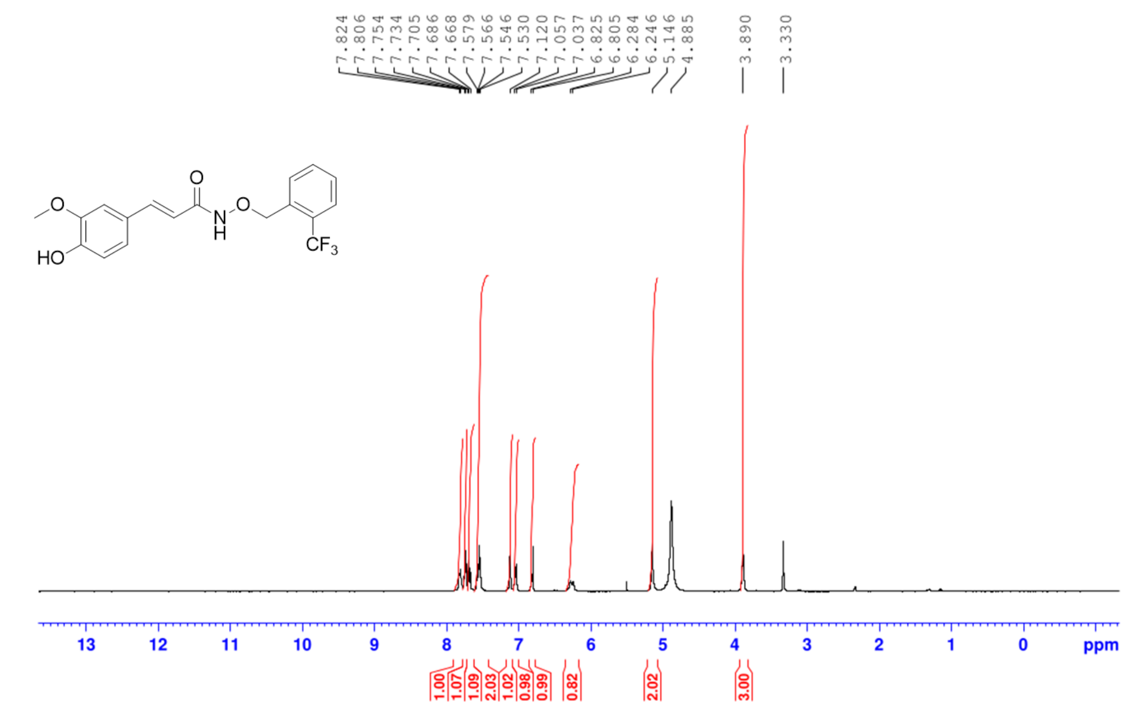

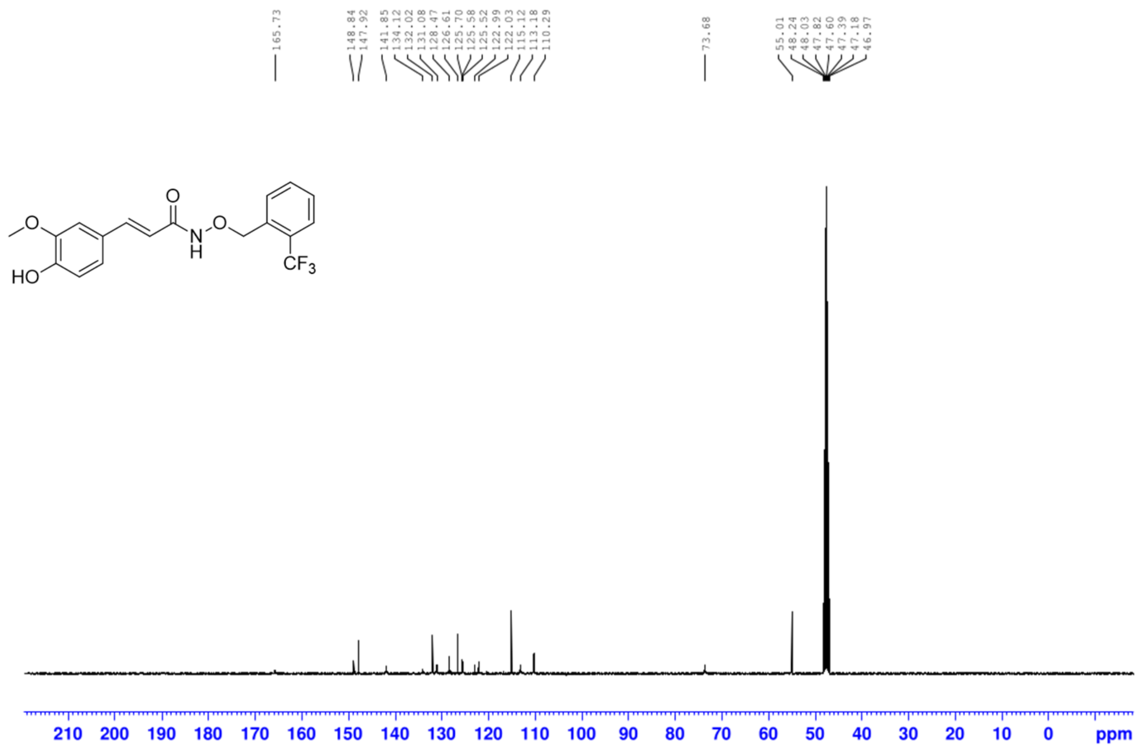

¹H and **¹³C NMR (MeOD)** spectra of compound **1d**.

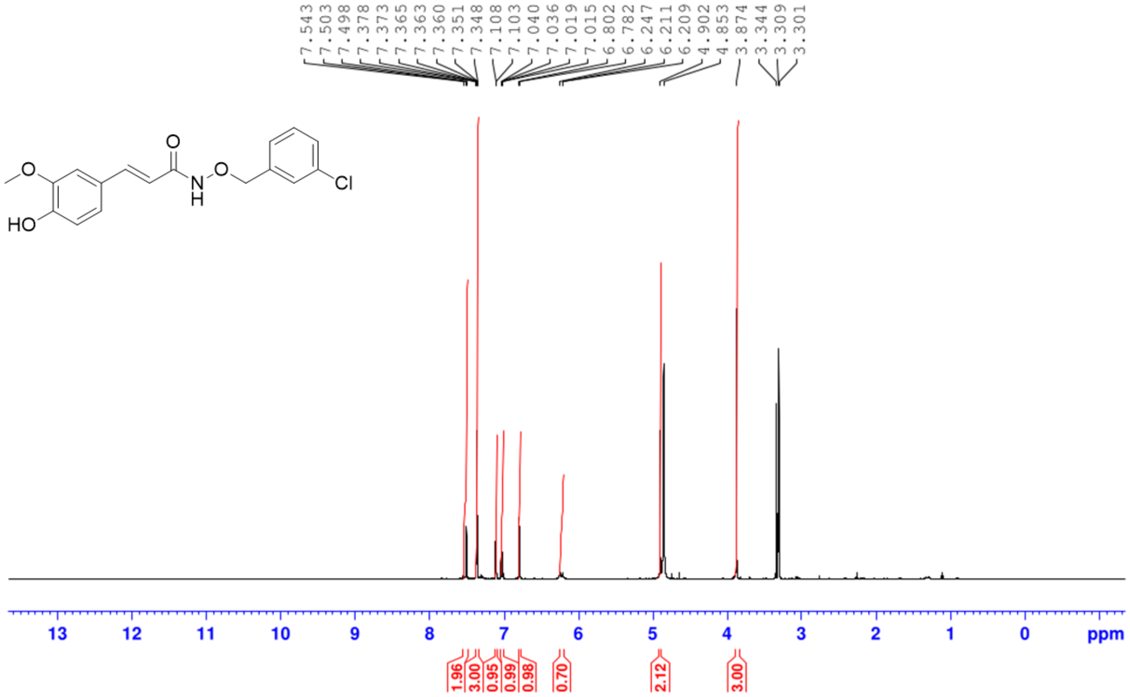

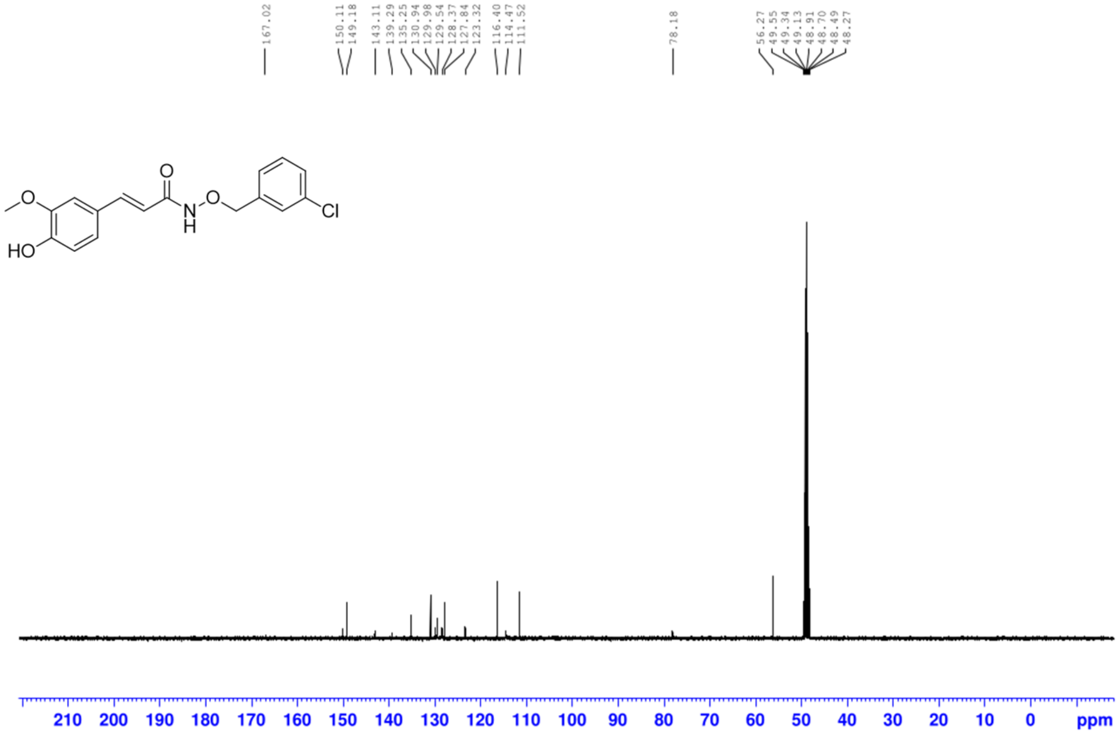

^{1}H and ^{13}C NMR (MeOD) spectra of compound **1e**.

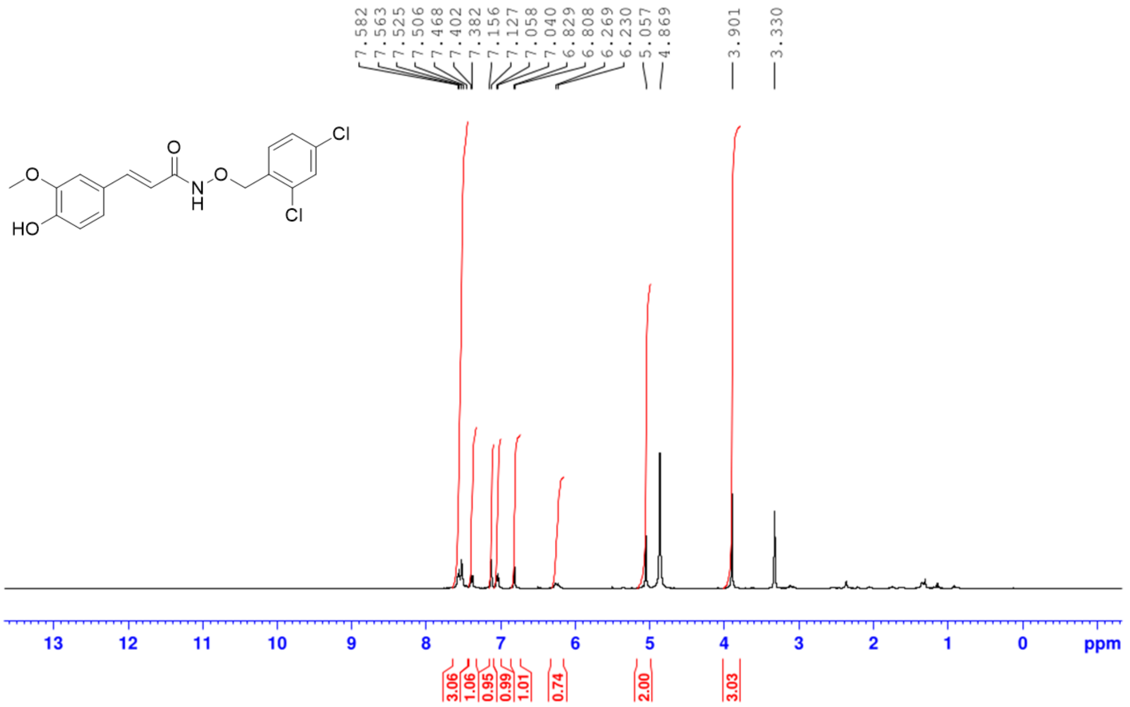

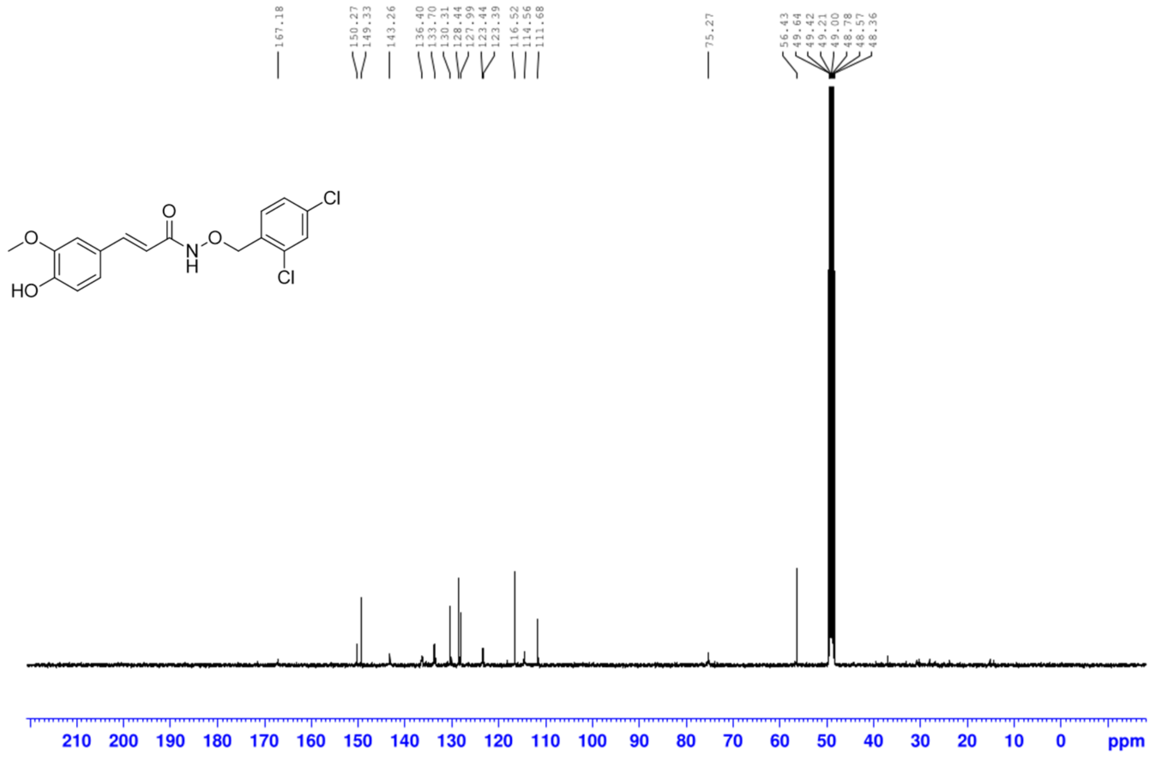

ESI-MS: $[M+H]^+$ compound **1a**.

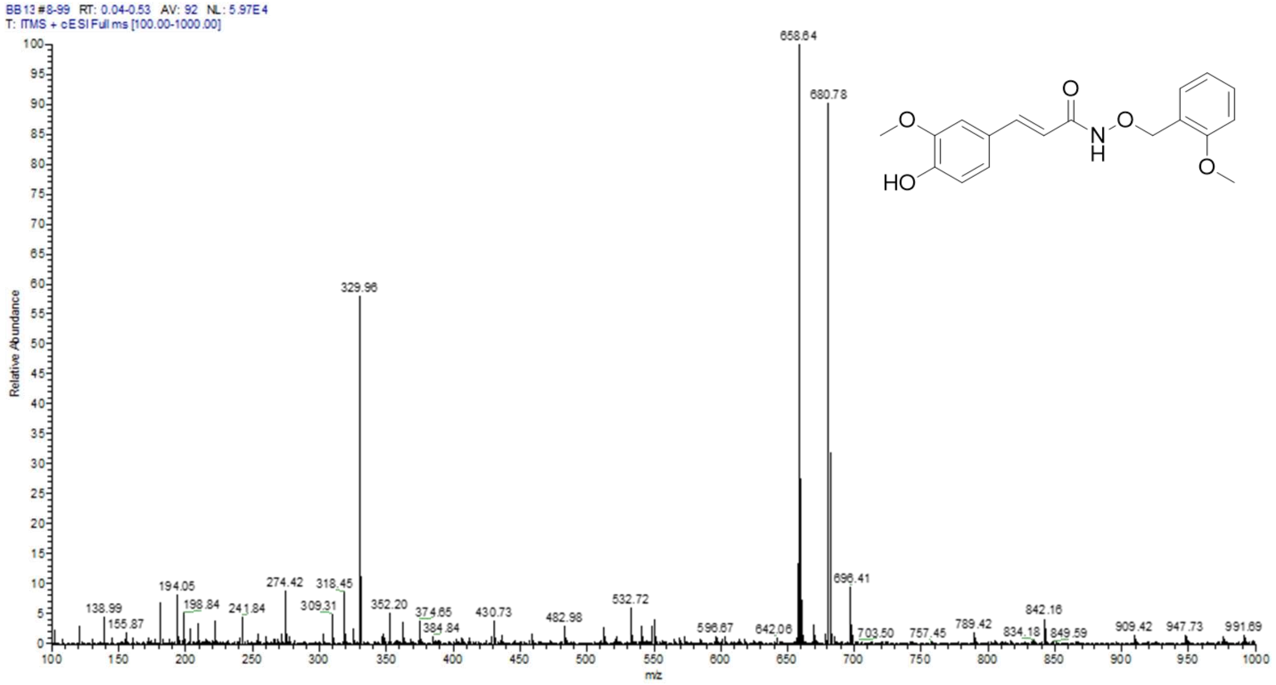

ESI-MS: $[M+H]^+$ compound **1b**.

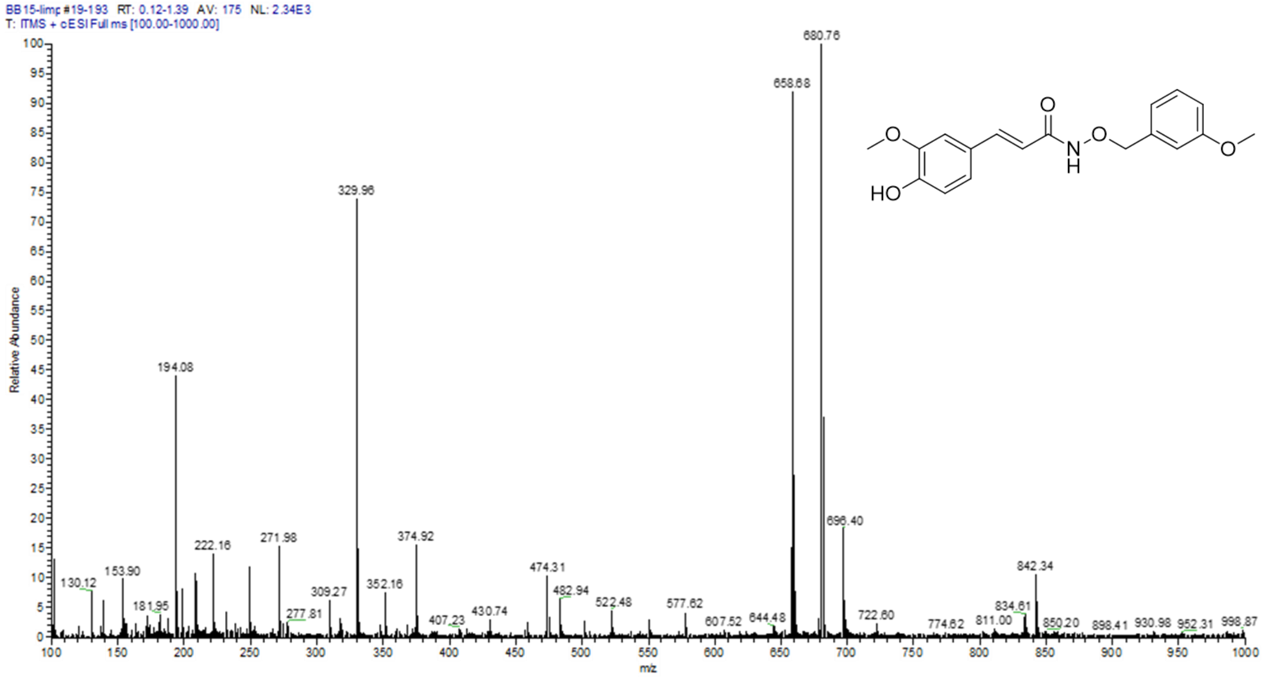

ESI-MS: $[M+H]^+$ compound **1c**.

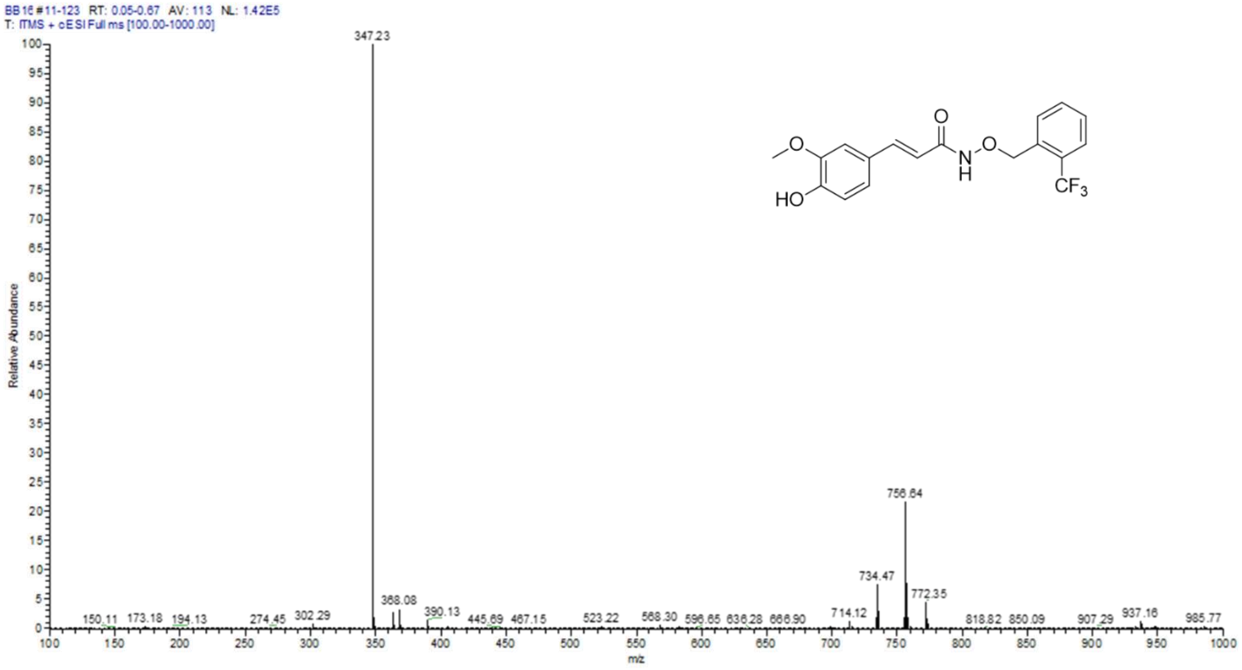

ESI-MS: $[M+H]^+$ compound **1d**.

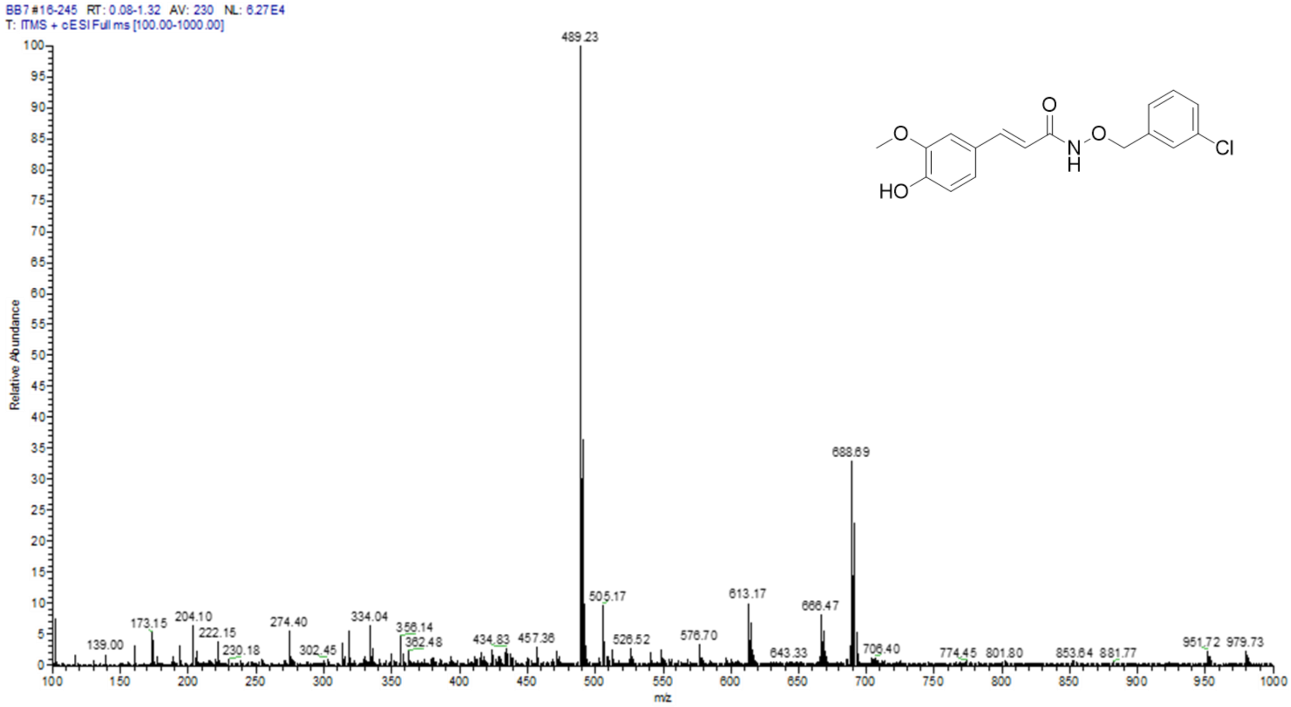

ESI-MS: [M+H]$^+$ compound **1e**.

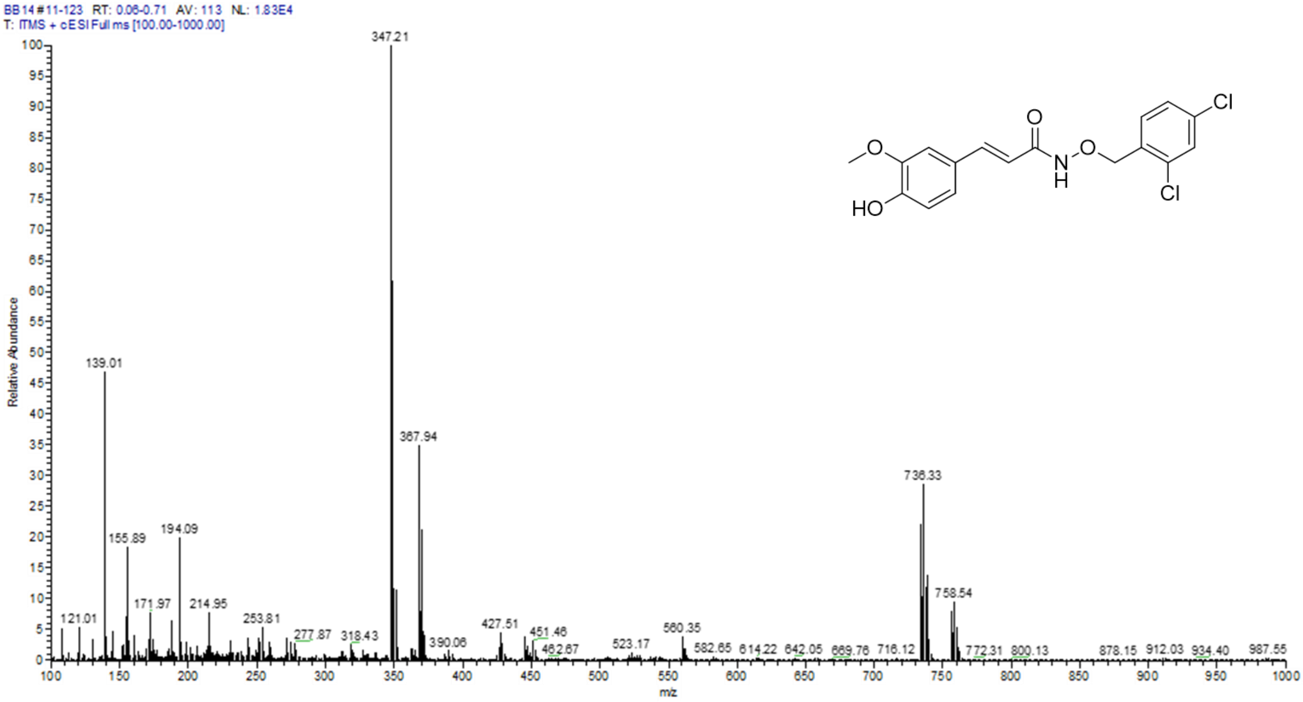

Supplement: Supplementary file 1 [file molecules-26-06015-s001.zip › molecules-1311149-supplementary.pdf]
